# Use of guidelines, checklists, frameworks, and recommendations in behavioral intervention preliminary studies and associations with reporting comprehensiveness: a scoping bibliometric review

**DOI:** 10.1186/s40814-023-01389-w

**Published:** 2023-09-13

**Authors:** Christopher D. Pfledderer, Lauren von Klinggraeff, Sarah Burkart, Alexsandra da Silva Bandeira, Bridget Armstrong, R. Glenn Weaver, Elizabeth L. Adams, Michael W. Beets

**Affiliations:** 1 Department of Health Promotion and Behavioral Sciences, The University of Texas Health Science Center at Houston, School of Public Health Austin Campus, Austin, TX 78701 USA; 2 Arnold School of Public Health, University of South Carolina, 921 Assembly Street, Columbia, SC 29205 USA

**Keywords:** Preliminary studies, Pilot, Feasibility, Framework, Review, Reporting quality

## Abstract

**Background:**

Guidelines, checklists, frameworks, and recommendations (GCFRs) related to preliminary studies serve as essential resources to assist behavioral intervention researchers in reporting findings from preliminary studies, but their impact on preliminary study reporting comprehensiveness is unknown. The purpose of this study was to conduct a scoping bibliometric review of recently published preliminary behavioral-focused intervention studies to (1) examine the prevalence of GCFR usage and (2) determine the associations between GCFR usage and reporting feasibility-related characteristics.

**Methods:**

A systematic search was conducted for preliminary studies of behavioral-focused interventions published between 2018 and 2020. Studies were limited to the top 25 journals publishing behavioral-focused interventions, text mined to identify usage of GCFRs, and categorized as either not citing GCFRs or citing ≥ 2 GCFRs (Citers). A random sample of non-Citers was text mined to identify studies which cited other preliminary studies that cited GCFRs (Indirect Citers) and those that did not (Never Citers). The presence/absence of feasibility-related characteristics was compared between Citers, Indirect Citers, and Never Citers via univariate logistic regression.

**Results:**

Studies (*n* = 4143) were identified, and 1316 were text mined to identify GCFR usage (*n* = 167 Citers). A random sample of 200 studies not citing a GCFR were selected and categorized into Indirect Citers (*n* = 71) and Never Citers (*n* = 129). Compared to Never Citers, Citers had higher odds of reporting retention, acceptability, adverse events, compliance, cost, data collection feasibility, and treatment fidelity (*OR*_range_ = 2.62–14.15, *p* < 0.005). Citers also had higher odds of mentioning feasibility in purpose statements, providing progression criteria, framing feasibility as the primary outcome, and mentioning feasibility in conclusions (*OR*_range_ = 6.31–17.04, *p* < 0.005) and lower odds of mentioning efficacy in purpose statements, testing for efficacy, mentioning efficacy in conclusions, and suggesting future testing (*OR*range = 0.13–0.54, *p* < 0.05). Indirect Citers had higher odds of reporting acceptability and treatment fidelity (*OR*_range_ = 2.12–2.39, *p* < 0.05) but lower odds of testing for efficacy (*OR* = 0.36, *p* < 0.05) compared to Never Citers.

**Conclusion:**

The citation of GCFRs is associated with greater reporting of feasibility-related characteristics in preliminary studies of behavioral-focused interventions. Researchers are encouraged to use and cite literature that provides guidance on design, implementation, analysis, and reporting to improve the comprehensiveness of reporting for preliminary studies.

**Supplementary Information:**

The online version contains supplementary material available at 10.1186/s40814-023-01389-w.

## Background

Early-stage, preliminary studies (i.e., pilot/feasibility) are the foundation of larger-scale behavioral interventions. Preliminary studies provide critical evidence regarding *trial feasibility* — recruitment and retention of participants and measurement of outcomes; *intervention feasibility* — participant enjoyment/acceptability, attendance/dosage, and missing or needed intervention components; and *preliminary efficacy* — whether changes are observed in primary or secondary outcomes to determine if an investment in a larger-scale, well-powered intervention is warranted [1, 2]. Particularly for preliminary studies, the capturing and comprehensive reporting of both trial and intervention feasibility are critical procedures needed to understand if the research can be appropriately conducted and whether the scale-up to a larger, more well-powered trial is worth pursuing. Furthermore, the thorough and transparent reporting of this information in published manuscripts may aid other researchers planning to conduct similar studies in learning how to design and implement successful preliminary studies of behavioral interventions.

Published guidelines, checklists, frameworks, and recommendations (GCFRs) are an essential resource for researchers conducting behavioral interventions to aid in the collection and reporting of intervention studies. For example, the Consolidated Standards of Reporting Trials (CONSORT) statement [3], the Template for Intervention Description and Replication (TIDieR) checklist [4, 5], and the Standard Protocol Items: Recommendations for Interventional Trials (SPIRIT) statement [6, 7], each provide important guidance for key elements needing to appear in published clinical trials. Evidence suggests the adoption of these guidelines has resulted in an overall improvement in reporting of clinical trials [5, 7], but additional work should be undertaken to understand how preliminary study-specific guidelines impact the reporting of the smaller, foundational studies, which are conducted prior to the larger-scale clinical trial.

For preliminary studies, there are multiple resources describing what should be collected and reported. Collectively, these resources describe high-quality preliminary studies as those which primarily focus on feasibility and thoroughly report all aspects of feasibility-related characteristics, study design, and implementation. These include the CONSORT extension to randomized pilot and feasibility trials [8] and recommendations from Pearson et al. [9] and Bowen et al. [10] for feasibility testing. Whether the use of such GCFRs leads to the comprehensive reporting of preliminary studies, however, is unknown. Evidence from a historical scoping review of 600 obesity-related preliminary studies published between 1982 and 2020 [11] suggests citing common GCFRs (e.g., CONSORT extension to pilot and feasibility studies) is positively associated with a more thorough reporting of feasibility indicators and higher preliminary study reporting quality. Guidance on preliminary studies, however, has only recently been published (within the past 20 years), and the usage and impact of these publications on recently published preliminary study reporting and comprehensiveness are not well understood. Additionally, there have not been any investigations into how GCFR utilization impacts the comprehensiveness of preliminary study reporting from a broader range of behavioral-focused intervention topics, not just those targeting obesity. The purpose of this study is to conduct a scoping bibliometric review of recently published (2018–2021) behavioral-focused preliminary intervention studies to identify the application of GCFRs and their association with the reporting of feasibility indicators and other feasibility-related study characteristics.

## Methods

This scoping bibliometric review was conducted and reported according to the Preferred Reporting Items of Systematic Reviews and Meta-Analyses extension for Scoping Reviews (PRISMA-ScR) guidelines [12].

### Identification of guidelines, checklists, frameworks, and recommendations

An iterative and systematic search was conducted to identify preliminary study-related guidelines, checklists, frameworks, and recommendations. Known GCFRs were used as starting points. These included the CONSORT Extension for Pilot and Feasibility Studies [8], Medical Research Council guidance [13], and publications such as Bowen et al. [10], Pearson et al. [9], and Eldridge et al. [14]. Backward citation tracking was used to identify additional GCFRs, which involved searching reference lists of identified GCFRs to locate additional relevant publications. This iterative approach was conducted until saturation was reached, and no new GCFRs could be identified. This approach has been used in previous literature reviews to identify and consolidate theoretical approaches in implementation research [15] and guidance on complex population health interventions [16]. Additionally, two separate samples of over 600 pilot studies from other reviews [11, 17] were used to identify GCFRs via backward citation tracking. Finally, the EQUATOR Network [18] was searched for any additional GCFRs related to preliminary studies. In total, we identified 152 GCFRs which we classified into 10 domains related to preliminary studies: *adaptations*, *definitions of pilot and feasibility studies*, *design and interpretation*, *feasibility*, *implementation*, *intervention development*, *progression criteria*, *reporting*, *sample size*, and *scale-up*. The Supplementary file contains a full list of these publications.

### Preliminary study search strategy

A systematic literature search was conducted in PubMed/MEDLINE, Embase/Elsevier, EBSCOhost, and Web of Science online databases for preliminary behavioral intervention studies published from January 1, 2018, to December 31, 2021. This time frame was chosen to highlight the most recently published behavioral intervention preliminary studies and to adequately capture usage of the most recently published GCFRs. The initial search strategy consisted only of the keywords as follows: “pilot,” “feasibility,” “preliminary,” “proof of concept,” and “vanguard” paired with the keyword “intervention” as found in the title and/or abstract. The following additional filters were applied to each search: English language, human species, articles only, peer-reviewed journals. A full search strategy is provided in the Supplementary File.

### Eligibility criteria and sampling strategy

Before the initial screening, the full list of articles was purposively sampled only from journals that served as likely outlets for published behavioral interventions. This was done to systematically reduce the large number of articles needed to be screened and focus on studies that were likely behavioral interventions. The full list of included journals can be found in the Supplementary file.

All preliminary studies published between 2018 and 2021 that delivered a behavioral intervention were considered for inclusion in this review. Similar to previous reviews [11, 17], behavioral interventions were defined as “interventions that target actions which lead to improvements in health indicators, separate from mechanistic, laboratory, pharmacological, feeding/dietary supplementation, and medical device or surgical procedure studies,” and preliminary studies were defined as “those studies which are conducted separately from and prior to a large-scale trial and are designed to test the feasibility of an intervention and/or provide evidence of preliminary effects before scale-up.” Articles were excluded if they employed a nonbehavioral intervention, were not a preliminary study, were only observational/cross-sectional in nature, or were presented only qualitative data.

### Screening process

Database search results were electronically downloaded as a RIS file and converted to an XML file, using EndNote X9 Reference Manager (Philadelphia, PA, USA), and uploaded to Microsoft Excel for review. The total number of articles downloaded from database search results was cross-checked with the total number of articles in the RIS and XML file to ensure all articles were converted to each file format. The XML file contained study information such as year, author, title, abstract, and the journal in which each article was published. Using the journal information, an article count for each journal was created, and the list of journals was sorted by article count. Journals that were not likely to have published a behavioral intervention were excluded from the list and their associated articles not considered for title and abstract screening. Articles published in the 25 journals with the largest article count were considered for screening. It is worth noting that all 25 journals endorsed the use of reporting guidelines when appropriate for the study type. Title and abstract screening were completed by three reviewers (C. D. P., L. V., A. B.) to identify remaining references that met the eligibility criteria. Disagreements were resolved by having a third member of the research team (M. B.) review the reference and make a final decision. Full-text PDFs were retrieved for references that passed the initial title and abstract screening process and were reviewed in duplicate by three members (C. D. P., L. V., A. B.) of the research team. Disagreements were resolved by having a third member of the research team (MWB) independently review the full-text manuscript and make a final decision.

### Study coding and categorization

The full list of studies that remained after the screening process was uploaded to NVivo 12 Plus Qualitative Data Analysis software (QSR International, 2021) as PDF files. To determine GCFR usage within each preliminary study, reference lists within each PDF were text mined in duplicate by two members of the research team (C. D. P. and L. V.) using the title of each GCFR identified from the search previously described. The presence or absence of a GCFR was documented for each study and each GCFR. An Excel matrix indicating the presence (coded as 1) or absence (coded as 0) of each GCFR for each preliminary study was exported from NVivo, and a sum total of cited GCFRs was created in a new column of the spreadsheet. Studies were initially categorized based on this sum total as “citers” (citing two or more GCFRs) and “Non-Citers” (citing no GCFRs). A copy of each file from both reviewers (C. D. P. and L. V.) was merged and cross-checked to ensure there were no discrepancies in data extraction and coding.

Because the non-Citers category had a large number of articles (*n* = 826), a random sample of 200 (~25%) were chosen for final inclusion in the review. All articles coded as non-Citers were assigned a unique, random identification number with STATA’s “rannum” command, sorted numerically, and the first 200 studies were included. After randomization, the non-Citers were further categorized as Never Citers, or Indirect Citers, using backward citation tracking and are defined below. Reference lists of the non-Citers were searched for (1) a protocol or preliminary study of the same intervention conducted by the same author, (2) a protocol or preliminary study of a different intervention conducted by the same author, or (3) a protocol or preliminary study conducted by different authors. If any of these were found in the non-Citers’ reference lists, the reference list of the cited study was searched for the presence/absence of GCFRs in the same way as the original sample. If a GCFR was present in the cited study’s reference list, the original Non-Citer was categorized as an Indirect Citer. If GCFRs were not present in any of the cited studies’ reference lists, the original Non-Citer was categorized as a Never Citer.

### Data extraction and coding

#### Descriptive characteristics

Study- and participant-level characteristics were extracted by four members of the research team (C. D. P., L. V., A. B., and J. P.) and coded in an Excel spreadsheet. These included publication year, location of study (country), baseline sample size, participant age, intervention treatment length, study design, trial registration information, and type of funding. An initial training set of 25 articles were coded in duplicate and cross-checked for consistency before the remaining articles were divided amongst the extraction team and extracted individually.

#### Reporting of feasibility indicators

The presence/absence of nine feasibility indicators was cataloged for each included preliminary study. Feasibility indicators included recruitment, retention, participant acceptability (satisfaction), adverse events, attendance, compliance, cost, data collection feasibility, and treatment fidelity. The list of relevant feasibility indicators was established in a previous review [11] and was based on definitions from the NIH and other peer-reviewed sources [19]. The identification of feasibility indicators was done by utilizing a combination of text mining and manual search procedures. Text mining procedures were conducted in NVivo 12 Plus Qualitative Data Analysis software (QSR International, 2021) by two members of the research team (C. D. P. and L. V.) and consisted of full-text searches with keywords related to feasibility outcomes. Full-text PDFs were also manually searched by CDP and LV to ensure text mining procedures identified all possible reported feasibility indicators. The presence or absence of each feasibility indicator was coded as 1 = present or 0 = absent for each included preliminary study. Similar to procedures used to extract descriptive characteristics, an initial training set of 25 articles were coded in duplicate by C. D. P. and L. V. and cross-checked for consistency before the remaining articles were divided amongst the extraction team and extracted/coded individually. Additional spot-checking was independently performed by a third member of the research team (M. B.), prior to analyses, to ensure data was accurately extracted. Examples of keywords used in the text mining procedures and the operational definitions of each feasibility indicator are provided in the Supplementary file.

#### Feasibility-related characteristics

The presence or absence of several feasibility-related characteristics was manually extracted. These included whether (1) the title included feasibility-related language (had “pilot,” “feasibility,” or similar language in the title), (2) the purpose statement mentioned feasibility and/or efficacy, (3) progression criteria were provided, (4) feasibility was framed as the primary outcome, (5) the conclusion mentioned feasibility and/or efficacy, (6) caution was advised regarding interpretations of preliminary efficacy, and (7) future testing was suggested. The presence or absence of each of these characteristics was similarly coded as 1 = present or 0 = absent. These feasibility-related characteristics, in addition to the reporting of feasibility indicators, were of interest because they are also important considerations unique to preliminary studies according to much of the guiding literature (8-10).

### Statistical analysis

Descriptive statistics were compared between Citers and the full sample (the total number of articles which constituted the larger sampling pool from which Never Citers, Indirect Citers, and Citers came from) as well as between Never Citers, Indirect Citers, and Citers using Kruskal-Wallis tests and chi-square tests when appropriate. Tests conducted between Citers and the full sample were conducted to ensure the quasi-random sampling procedure used to select studies did not produce any systematic differences between groups. Univariate logistic regression models were employed to assess differences in feasibility-related characteristics and in the reporting of feasibility indicators between Never Citers, Indirect Citers, and Citers. The presence or absence of feasibility-related characteristics and feasibility indicators was treated as the binary dependent variable, while two dummy variables identifying Indirect Citers and Citers (with Never Citers as the reference category) were independent variables. Because we did not consider the original CONSORT [3] a true preliminary study-related GCFR, we accounted for its presence by including its use as a covariate in all univariate models (coded as 0 = not cited, 1 = cited). Additionally, for four of the feasibility-related characteristics (mentioning feasibility in the conclusion, mentioning efficacy in the conclusion, advising caution regarding efficacy, suggesting future testing), we did not include protocols in the sample used for the logistic regression models, as these aspects of feasibility would not have been reported in protocol studies. All analyses had an alpha level of *p* < 0.05 and were carried out using STATA v17.0 statistical software package (College Station, TX, USA).

## Results

### Search results

**Fig. 1:**
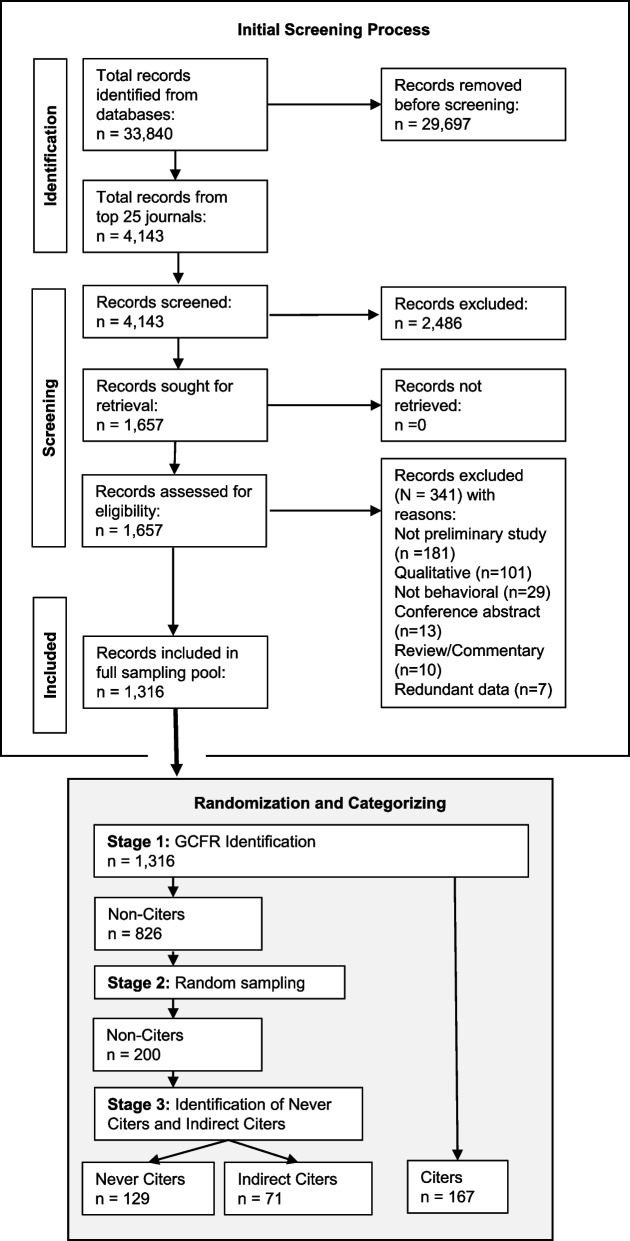
Preferred Reporting Items for Systematic Reviews and Meta-Analyses (PRISMA) consort diagram

Results of the screening and randomization procedures are communicated in the study flow diagram (Fig. 1). A total of 33,840 records were identified from the database search. Limiting records to only the 25 health behavior journals with the largest article counts reduced the number of records to 4143. Abstract screening further reduced this sample to 1657. After assessing articles for eligibility, a total of 341 were excluded, and 1316 articles were included in the full sample

The full sample of 1316 articles was then text mined to identify the usage of GCFRs within their references. A total of 826 articles cited zero GCFRs, and 167 cited two or more. A random sample of 200 articles from the 826 which cited zero GCFRs were further categorized into Never Citers (*n* = 129) and Indirect Citers (*n* = 71) using the methodology previously described. Thus, a total of 367 articles were included in this bibliometric review.

### Descriptive characteristics

Table 1 communicates descriptive statistics for all studies included in the bibliometric review and separately for Never Citers, Indirect Citers, and Citers. For the included sample (*N* = 367), most studies were conducted in Europe (*n* = 135, 36.8%) or North America (*n* = 133, 36.2%), targeted adults (*n* = 290, 79.0%), had a treatment length of 12 weeks or less (*n* = 246, 69.4%), and reported funding of some kind (*n* = 320, 87.2%). Most studies employed either a RCT (*n* = 156, 42.5%) or a non-randomized, single-group design (*n* = 142, 38.7%), and 210 (57.2%) were registered trials. A total of 126 (34.3%) studies were protocols.

**Table 1:** Characteristics of included preliminary studies (*N* = 367)

**Characteristics**	**Included sample**		**Never citers**		**Indirect citers**		**Citers**	
	*N* = 367		*N* = 129		*N* = 71		*N* = 167	
	*N*	Percent (%)	*N*	Percent (%)	*N*	Percent (%)	*N*	Percent (%)
**Year**								
2018	77	20.9	34	26.4	15	21.1	28	16.8
2019	96	26.2	36	27.9	17	23.9	43	25.7
2020	106	28.9	35	27.1	20	28.2	51	30.5
2021	88	23.9	24	18.6	19	26.8	45	26.9
**Location**								
Africa	21	5.7	10	7.8	6	8.5	5	2.9
Asia	30	8.2	19	14.7	6	8.5	5	2.9
Europe	135	36.8	32	24.8	15	21.1	88	52.7
North America	133	36.2	56	43.4	32	45.1	45	26.9
Oceania	43	11.7	11	8.5	11	15.5	21	12.6
South America	2	0.5	1	0.8	1	1.4	0	0.0
Multi-continent	3	0.8	0	0.0	0	0.0	3	1.8
**Baseline sample size (*N*)**								
1–24	68	22.4	21	17.9	20	31.8	27	21.9
25–49	91	30.0	41	35.0	15	23.8	35	28.5
50–99	72	23.7	26	22.2	11	17.5	35	28.5
100 or more	72	23.7	29	24.8	17	26.9	26	21.1
**Participant age**								
Youth	37	10.1	16	12.4	10	14.1	11	6.6
Adult	290	79.0	98	75.9	54	76.1	138	82.6
Both	40	10.9	15	11.6	7	9.9	18	10.8
**Treatment length (weeks)**								
6 weeks or less	101	28.5	45	36.6	21	30.4	35	21.5
7–12 weeks	145	40.9	41	33.3	30	43.5	74	45.4
13–26 weeks	71	20.0	26	21.1	12	17.4	33	20.3
27–52 weeks	28	7.9	8	6.5	3	4.4	17	10.4
53 weeks or more	10	2.8	3	2.4	3	4.4	4	2.5
**Design**								
Randomized controlled trial	156	42.5	51	39.5	24	33.8	81	48.5
Non-randomized, single group	142	38.7	54	41.9	33	46.5	55	32.9
Non-randomized, multi-intervention	41	11.2	18	13.9	10	14.1	13	7.8
Randomized, multi-intervention	28	7.6	6	4.7	4	5.6	18	10.8
**Protocols**	126	34.3	29	22.5	12	16.9	85	50.9
**Registered trials**	210	57.2	49	37.9	46	64.8	131	78.4
**Funding**								
Any	320	87.2	107	82.9	62	87.3	151	90.4
CIHR (Canada)	7	2.2	2	1.9	2	3.2	3	1.9
MRC (UK)	9	2.8	4	3.7	1	1.6	4	2.6
NIH (USA)	91	28.4	33	30.8	19	30.6	39	25.8
NIHR (Europe)	46	14.4	10	9.3	10	16.1	26	17.2
NHMRC (Australia)	12	3.8	5	4.7	3	4.8	4	2.6

### Usage of guidelines, checklists, frameworks, and recommendations

Table 2 displays the list of cited GCFRs by topic, with citation counts for the full sample (*N* = 1316) and for Citers (*N* = 167). The Supplementary file contains a full list of GCFRs that were searched for within the references of the studies in the full sample. For the full sample (*N* = 1316), the top three cited domains were *reporting* (*n* = 281, 21.4%), *design and interpretation* (*n* = 269, 20.4%), and *definitions of pilot and feasibility studies* (142, 10.8%). Table 3 displays the top ten cited GCFRs. Briefly, the top five GCFRs were Craig et al. [13, 20] (*n* = 128, 9.7%), Eldridge et al. [8] (*n* = 123, 9.3%), Hoffman et al. [21] (*n* = 79, 6.0%), Bowen et al. [10] (*n* = 63, 4.8%), and Thabane [22] (*n* = 62, 4.6%).

**Table 2:** List of GCFR topics with citation counts for the full sample and those in the Citers category

**GCFR topic**	**Full sample** (*n* = 1316)		**Citers** (*n* = 167)	
	**Citation count**	**Percent (%)**	**Citation count**	**Percent (%)**
Adaptations	27	2.1	15	8.9
Defining pilot and feasibility studies	142	10.8	76	45.5
Design and interpretation	269	20.4	129	77.2
Feasibility	97	7.3	50	29.9
Guidance review	2	0.2	1	0.6
Implementation	123	9.3	50	29.9
Intervention development	35	2.7	17	10.2
Progression criteria	16	1.2	9	5.4
Reporting	281	21.4	113	67.7
Sample size/power	76	5.8	31	18.6
Scale-up	30	2.3	13	7.8

**Table 3:** List of the top 10 most cited GCFRs

**GCFR**	**Topic**	**Full sample** (*n* = 1316)		**Citers** (*n* = 167)	
		**Citation count**	**Percent (%)**	**Citation count**	**Percent (%)**
Craig [13, 20]	Design and interpretation	128	9.7	65	38.9
Eldridge [23] (CONSORT ext.)	Reporting	123	9.3	35	20.9
Hoffmann [24]	Reporting	79	6.0	43	25.7
Bowen [25]	Feasibility	63	4.8	27	16.2
Thabane [26–28]	Reporting	62	4.6	37	22.2
Lancaster [29]	Design and interpretation	56	4.3	33	19.8
RE-AIM [30]	Implementation	50	9.8	21	12.6
Leon [31]	Design and interpretation	43	3.3	10	5.9
Eldridge [32] (defining)	Defining pilot and feasibility studies	39	2.9	18	10.8
Chan [33]	Reporting	32	2.4	12	7.2
Billingham [34]	Sample size/power	32	2.4	18	10.8

### Reporting of feasibility indicators

Table 4 summarizes the reporting of feasibility indicators for all studies included in the review and separately for Never Citers, Indirect Citers, and Citers. For the included sample (*N* = 367), recruitment (*n* = 281, 76.6%), retention (*n* = 278, 75.8%), and participant acceptability (*n* = 262, 71.4%) were the most common feasibility indicators reported. Participant compliance (*n* = 61, 16.6%), data collection feasibility (*n* = 56, 15.3%), and cost (*n* = 39, 10.6%) were the least common feasibility indicators reported. In terms of the total number of feasibility indicators reported in the total sample of preliminary studies, 57 (15.5%) reported none, 75 (20.4%) reported one, 93 (25.3%) reported two, 71 (19.4%) reported three, and 71 (19.4%) reported four or more.

**Table 4:** Feasibility-related characteristics of included preliminary studies (*N* = 367)

**Feasibility-related characteristics**	**Included sample**		**Never Citers**		**Indirect Citers**		**Citers**	
	*N* = 367		*N* = 129		*N* = 71		*N* = 167	
	*N*	Percent (%)	*N*	Percent (%)	*N*	Percent (%)	*N*	Percent (%)
**Feasibility indicators**								
Recruitment	281	76.6	91	70.5	47	66.2	143	85.6
Retention	278	75.8	80	62.0	51	71.8	147	88.0
Acceptability	262	71.4	64	49.6	50	70.4	148	88.6
Adverse events	163	44.4	42	32.6	28	39.4	93	55.7
Attendance	66	17.9	11	8.5	8	11.3	47	28.1
Compliance	61	16.6	18	13.9	14	19.7	29	17.4
Cost	39	10.6	6	4.7	3	4.2	30	17.9
Data collection	56	15.3	9	6.9	5	7.0	42	25.2
Treatment fidelity	136	37.1	20	15.5	20	28.2	96	57.5
**Total outcomes reported**								
0	57	15.5	40	31.0	12	16.9	5	2.9
1	75	20.4	39	30.2	20	28.2	16	9.6
2	93	25.3	27	20.9	24	33.8	42	25.2
3	71	19.4	17	13.2	3	4.2	51	30.5
4 or more	71	19.4	6	4.7	12	16.9	53	31.7
**Feasibility-related characteristics**								
Title includes feasibility-related language	279	76.0	93	72.0	46	64.8	140	83.8
Feasibility mentioned in purpose statement	274	74.7	74	57.4	46	64.8	154	92.2
Efficacy mentioned in purpose statement	204	55.6	81	62.8	40	56.3	83	49.7
Progression criteria provided	45	12.3	2	1.6	2	1.8	41	24.6
Statistical testing for efficacy	246	67.0	112	86.8	50	70.4	84	50.3
Feasibility framed as primary outcome	267	72.8	70	54.3	44	61.9	153	91.6
Feasibility of the study mentioned in conclusion^a^	180	74.7	62	62.0	45	76.3	73	89.0
Efficacy of the study mentioned in conclusion^a^	134	55.6	64	64.0	32	54.2	38	46.3
Caution advised regarding efficacy^a^	130	53.9	58	58.0	36	61.0	36	43.9
Future testing suggested^a^	216	89.6	95	95.0	53	89.8	68	82.9

^a^Protocols were not included in this analysis. See Table 1 for total number of protocols in each citation category

Results from univariate logistic regression models for the presence or absence of feasibility indicators are presented in Table 5. Compared to Never Citers, Citers were significantly more likely to report all feasibility indicators except for recruitment and attendance. These included retention (*OR* = 3.75, 95% *CI*: 1.98–7.13), participant acceptability (*OR* = 14.15, 95% *CI*: 6.43–31.15), adverse events (*OR* = 4.38, 95% *CI*: 2.07–9.23), participant compliance (*OR* = 2.62, 95% *CI*: 1.55–4.45), cost (*OR* = 4.35, 95% *CI*: 1.66–11.40), data collection (*OR* = 4.35, 95% *CI*: 1.93–9.78), and treatment fidelity (*OR* = 8.82, 95% *CI*: 4.73–16.44). Compared to Never Citers, Indirect Citers were more likely to report participant acceptability outcomes (*OR* = 2.39, 95% *CI*: 1.29–4.45) and outcomes related to treatment fidelity (*OR* = 2.12, 95% *CI*: 1.05–4.29).

**Table 5:** Summary of logistic regression analysis for reporting feasibility indicators and other related characteristics in studies considered Never Citers, Indirect Citers, and Citers (*N* = 367)

Feasibility-related reporting characteristics	Indirect Citers^a^			Citers^a^		
	*N* = 71			*N* = 167		
	Odds ratio	95% *CI*^b^	*p*-value	Odds ratio	95% *CI*^b^	*p*-value
*Feasibility indicators*						
Recruitment	0.83	0.44–1.54	0.544	1.78	0.97–3.28	0.065
Retention	1.58	0.84–2.96	0.155	**3.75**	**1.98**–**7.13**	**< 0.001**
Acceptability	**2.39**	**1.29**–**4.45**	**0.006**	**14.15**	**6.43**–**31.15**	**< 0.001**
Adverse events	1.36	0.52–3.55	0.531	**4.38**	**2.07**–**9.23**	**< 0.001**
Attendance	1.51	0.70–3.25	0.293	1.39	0.69–2.79	0.358
Compliance	1.35	0.74–2.46	0.330	**2.62**	**1.55**–**4.45**	**< 0.001**
Cost	0.91	0.22–3.73	0.891	**4.35**	**1.66**–**11.40**	**0.003**
Data collection	1.01	0.33–3.14	0.984	**4.35**	**1.93**–**9.78**	**< 0.001**
Treatment fidelity	**2.12**	**1.05**–**4.29**	**0.036**	**8.82**	**4.73**–**16.44**	**< 0.001**
*Feasibility-related characteristics*						
Title includes feasibility-related language	0.72	0.39–.34	0.303	1.51	0.82–2.78	0.188
Feasibility mentioned in purpose statement	1.36	0.75–2.48	0.314	**9.87**	**4.58**–**21.23**	**< 0.001**
Efficacy mentioned in purpose statement	0.77	0.43–1.38	0.379	**0.54**	**0.32**–**0.91**	**0.020**
Progression criteria provided	1.86	0.26–13.51	0.539	**17.04**	**3.91**–**74.29**	**< 0.001**
Statistical testing for efficacy	**0.36**	**0.18**–**0.75**	**0.006**	**0.13**	**0.07**–**0.26**	**< 0.001**
Feasibility framed as primary outcome	1.37	0.76–2.48	0.296	**9.66**	**4.63**–**20.17**	**< 0.001**
Feasibility of the study mentioned in conclusion^c^	1.95	0.95–4.03	0.070	**6.31**	**2.43**–**16.36**	**< 0.001**
Efficacy of the study mentioned in conclusion^c^	0.67	0.35–1.28	0.223	**0.50**	**0.26**–**0.99**	**0.046**
Caution advised regarding efficacy^c^	1.13	0.59–2.18	0.716	0.61	0.31–1.19	0.152
Future testing suggested^c^	0.46	0.33–1.59	0.223	**0.26**	**0.08**–**0.84**	**0.024**

^a^Never Citers (*N* = 129) is the referent group

^b^95% CI stands for 95% confidence interval

^c^Protocols excluded from analysis

Bold denotes significance at the *p* < 0.05 level

### Feasibility-related characteristics

Table 4 also summarizes the presence of feasibility-related characteristics for all studies included in the review and separately for Never Citers, Indirect Citers, and Citers. For the included sample (*N* = 367), 279 (76.0%) had feasibility-related language in the title, and 274 (74.7%) mentioned feasibility in the purpose statement of the study. Feasibility was framed as the primary outcome in 267 (72.8%) studies. Conversely, 204 (55.6%) studies mentioned efficacy in the purpose statement, and 246 (67.0%) conducted statistical testing for efficacy. Only 45 (12.3%) provided progression criteria. For non-protocol studies (*n* = 241), 180 (74.7%) mentioned feasibility in the conclusion, while 134 (55.6%) mentioned efficacy in the conclusion. A total of 130 (53.9%) non-protocol studies advised caution when interpreting the preliminary efficacy of their intervention, and 216 (89.6%) suggested future testing of the intervention.

Results from univariate logistic regression models for the presence or absence of feasibility-related characteristics are also presented in Table 5. Compared to Never Citers, Citers were significantly more likely to mention feasibility in the purpose statement (*OR* = 9.87, 95% *CI*: 4.58–21.23), provide progression criteria (*OR* = 17.04, 95% *CI*: 3.91–74.29), frame feasibility as the primary outcome (*OR* = 9.66, 95% *CI*: 4.63–20.17), and mention the feasibility of the study in the conclusion (*OR* = 6.31, 95% *CI*: 2.43–16.36). Citers were significantly less likely to mention efficacy in the purpose statement (*OR* = 0.54, 95% *CI*: 0.32–0.91), conduct statistical testing for efficacy (*OR* = 0.13, 95% *CI*: 0.07–0.26), mention the efficacy of the study in the conclusion (*OR* = 0.50, 95% *CI*: 0.26–0.99), and suggest future testing of the intervention (*OR* = 0.26, 95% *CI*: 0.08–0.84). Compared to Never Citers, Indirect Citers were significantly less likely to conduct statistical testing for efficacy (*OR* = 0.36, 95% *CI*: 0.18–0.75).

## Discussion

This was a scoping bibliometric review of 367 behavioral-focused preliminary intervention studies published between 2018 and 2020. We examined the usage of guidelines, checklists, frameworks, and recommendations (GCFRs) related to preliminary studies and determined associations between GCFR usage and the reporting of feasibility indicators and feasibility-related characteristics. Citing two or more GCFRs was associated with reporting a greater number of feasibility indicators and framing the study findings from a feasibility perspective. These data demonstrate the use of GCFR, as inferred from citations within reference lists, and have a clear positive impact on the overall comprehensiveness of the information presented. This information, in turn, should lead to greater transparency in reporting and more informed decisions regarding the viability of a behavioral intervention in a larger-scale trial.

To the authors’ knowledge, this is the first review to document and analyze the usage of GCFRs in preliminary health behavior preliminary intervention studies. Literature on *reporting*, *design and interpretation*, and *defining pilot and feasibility studies* made up the majority of citations, and the three most cited GCFRs were the Medical Research Council (MRC) Guidance for Developing and Evaluating Complex Interventions [13, 20], the CONSORT Extension for Pilot and Feasibility Studies [8], and the Template for Intervention Description and Replication (TIDieR) [21]. According to the Web of Science (accessed April 14, 2022), the MRC guidance has been cited over 4000 times, the CONSORT extension over 500 times, and TIDieR over 3000 times, although preliminary studies do not account for all of these citations. It is not surprising though that these are the top cited GCFRs in our sample of preliminary studies as each of them are widely supported in the behavioral sciences, funding agencies. These GCFRs can also be found on the EQUATOR network [18], which makes them easily accessible to a larger audience of researchers developing intervention studies.

Less common were GCFRs related to *adaptations* and *scale-up*, with the least commonly cited GCFR category being *progression criteria*. This might explain the very low presence of progression criteria in our included sample of preliminary studies, which has been found in other reviews as well [22, 35]. The low usage of GCFRs related to scale-up is interesting, especially since the majority of preliminary studies are presumably designed for future scale-up. Several frameworks, including the National Institutes of Health Stage Model [36] and the Obesity-Related Behavioral Intervention Trials (ORBIT) model [37], outline the sequential and iterative processes for developing large-scale interventions, and specific attention is devoted to preliminary studies as a foundational piece of this scale-up process. Many of the GCFRs highlighted in this review can provide useful guidance on the scale-up process.

The reporting of feasibility indicators and feasibility-related characteristics in the sample of preliminary studies included in this review is similar to that of other reviews of health behavior intervention studies that are not preliminary studies [11, 35, 38]. Most studies did not report feasibility indicators such as treatment fidelity, data collection feasibility, cost, participant compliance and attendance, and adverse events. Overall, most studies reported two or fewer feasibility indicators, which were typically recruitment and retention of participants. While trial-related feasibility (i.e., recruitment and retention) is important to assess, intervention-related feasibility indicators (treatment fidelity, the ability to collect data on participants, etc.) are equally important to measure and report to highlight the potential viability of an intervention and/or what aspects of the intervention need to be addressed and altered to ensure the intervention is successfully scaled up in the future.

While most studies did mention feasibility (or use similar language) in the title and purpose statement, over half mentioned efficacy in the purpose statement and conducted statistical testing for efficacy. It is well-established researchers should exercise caution when conducting efficacy testing within preliminary intervention studies and should not depend on these estimates to inform the design of a larger-scale trial, which would likely be conducted with hypothesis testing in mind [39–41]. In fact, many of the GCFRs identified for this review caution against the use and interpretation of preliminary study effect sizes. This could explain why the use of GCFRs was associated with lower odds of many efficacy-related study characteristics in our sample. There are also inherent issues (both type I and type II error) with using preliminary study effect sizes for sample size estimation for a larger-scale trial, although we did not identify whether this was being done in our sample of preliminary studies. It is worth noting that just over half of the included preliminary studies did advise caution regarding interpretations of efficacy, and most studies framed feasibility indicators as the primary outcomes of interest. This is a promising finding and shows that many authors of recently published preliminary studies are [1] acknowledging the pitfalls of using preliminary studies to demonstrate the efficacy of an intervention and [2] prioritizing feasibility indicators as the main outcome of interest.

While this study is not the first to explore reporting quality in preliminary studies [11, 35, 38], it is the first to investigate associations between the usage of GCFRs and preliminary study reporting. Some studies have broadly explored the impact of guidelines (mostly CONSORT and TIDieR) on study reporting quality, not necessarily in preliminary studies exclusively, and the results of these studies are heterogeneous [42–44]. There are also several other examples of these types of reviews for a variety of disciplines and study designs [45–49], but most use somewhat ambiguous proxies for the usage of guidelines. In other words, most reviews have assessed reporting quality before and after the publication of a guideline, with the assumption that the presence of the published guideline might influence reporting quality. Our study utilized text mining to identify preliminary studies that cited GCFRs, using the presence of a GCFR citation as the indicator for usage and found that usage significantly and positively associated with the reporting of most feasibility indicators and feasibility-related characteristics. These findings provide compelling support for the use of GCFRs as a tool to improve the reporting quality of preliminary intervention studies.

Not only does the usage of GCFRs (via citation) associate with better reporting, our results in the Indirect Citer group, show citing a different preliminary study that cited a GCFR associated with increased odds of reporting acceptability and treatment fidelity as well as decreased odds of conducting statistical testing for efficacy. These results demonstrate a possible diffusive nature of the information published in GCFRs, whereby authors use other published preliminary studies (which cited a GCFR) as a model for their work. Thus, the true beneficial impact of GCFRs related to preliminary studies may go beyond just improving the reporting quality of studies which cite them.

### Strengths and limitations

This review included a large sample (*N* = 367) of preliminary intervention studies published between 2018 and 2020, capturing some of the most recently published health behavior preliminary studies in the literature. Preliminary studies were not excluded based on location, design, or health behavior topic, which means results are generalizable to a large audience of health behavior researchers who conduct preliminary intervention studies. However, several limitations need to be considered. Included studies came from a sample 25 journals publishing the largest number of preliminary studies. Sampling from a wider variety of journals may have produced different results. Because the number of studies not citing any GCFR (Non-Citers) was so large, we opted to randomly sample 200 of them. Although random sampling is supposed to be an unbiased approach to sampling and no differences between the subsample and larger sample were found in our study, there is a possibility of the presence of sample selection bias. Limitations regarding the coding of studies are also important to mention. First, the presence/absence of GCFR usage and the reporting of feasibility indicators and feasibility-related characteristics were identified via a combination of text mining and manual approaches. It is possible that some items may have either not been identified or improperly coded due to human error, although several quality control checks were put in place to avoid these issues. While we used an extensive list of GCFRs, it is possible that some GCFRs were missed and therefore not searched within our sample, which could influence results by miscoding studies that did cite a GCFR which we did not include in our search. Finally, we recognize that what is reported in a study is not completely identical to the actual conduct of the study. The failure to cite a GCFR or report a feasibility indicator does not mean authors of a study had not considered preliminary study guidance or measured feasibility indicators during their preliminary intervention study.

## Conclusions

Preliminary studies provide an ideal opportunity to improve multiple facets of an intervention study. Each of these improvements has the potential to enhance the overall rigor and reproducibility of the intervention when delivered at scale. Tools to aid researchers in the development and implementation of preliminary studies exist, namely in the form of published guidelines, checklists, frameworks, and recommendations, but our review indicates that many are never cited. Results from this review provide evidence that the use of GCFRs (via citation) is associated with more thorough reporting of feasibility-related characteristics in behavioral-focused preliminary studies. Researchers should utilize available literature that provides guidance on various aspects of preliminary study design, implementation, analysis, and reporting to improve the comprehensiveness and reporting of their preliminary studies, inform efficient scale-up to larger-scale trials, and avoid unnecessary research waste.

### Supplementary Information

**Additional file 1:** **Supplementary Table 1.** List of top 25 included journals and their citation count in the full sample, the subsample, Non-Citers, and Citers. **Supplementary Table 2.** Full list of guidelines, checklists, frameworks, and recommendations by topic with citation counts for the full sample and those in the Citers category. **Supplementary Table 3.** Operational definitions of trial- and intervention-related feasibility indicators and keywords used to search for them via text-mining.

## Data Availability

The datasets used and analyzed during the current study are freely available at https://osf.io/5sd28/.

## Abbreviations

CIHR: Canadian Institutes of Health Research
CONSORT: Consolidated Reporting of Trials
EQUATOR: Enhancing the QUAlity and Transparency Of health Research
GCFRs: Guidelines, checklists, frameworks, and recommendations
MRC: Medical Research Council
NHMRC: National Health and Medical Research Council
NIH: National Institutes of Health
NIHR: National Institutes of Health Research
OR: Odds ratio
ORBIT: Obesity-Related Behavioral Intervention Trials
PRISMA: Preferred Reporting Items for Systematic Reviews and Meta-Analyses
PRISMA-ScR: Preferred Reporting Items of Systematic Reviews and Meta-Analyses extension for scoping reviews
RCT: Randomized controlled trial
SPIRIT: Standard Protocol Items: Recommendations for Interventional Trials
TIDieR: Template for Intervention Description and Replication
