# Supplementary material for: Use of guidelines, checklists, frameworks, and recommendations in behavioral intervention preliminary studies and associations with reporting comprehensiveness: a scoping bibliometric review
Source: Pilot Feasibility Stud. 2023 Sep 13;9:161. doi: 10.1186/s40814-023-01389-w (PMC10498529; doi:10.1186/s40814-023-01389-w)
Supplement: Supplementary file 1 — Additional file 1: Supplementary Table 1. List of top 25 included journals and their citation count in the full sample, the subsample, Non-Citers, and Citers. Supplementary Table 2. Full list of guidelines, checklists, frameworks, and recommendations by topic with citation counts for the full sample and those in the Citers category. Supplementary Table 3. Operational definitions of trial- and intervention-related feasibility indicators and keywords used to search for them via text-mining. [file 40814_2023_1389_MOESM1_ESM.docx]

| **Journal** | **Citation Count in Full List (N=4,143)** | | **Citation Count in Included Sample (N=367)** | | **Citation Count for Non-Citers**  **(N=200)** | | **Citation Count for Citers**  **(N=167)** | |
| --- | --- | --- | --- | --- | --- | --- | --- | --- |
|  | **N** | **Percent (%)** | **N** | **Percent (%)** | **N** | **Percent (%)** | **N** | **Percent (%)** |
| Age and Ageing | 69 | 1.7 | 2 | 0.5 | 1 | 0.5 | 1 | 0.6 |
| AIDS and Behavior | 100 | 2.4 | 10 | 2.7 | 9 | 4.5 | 1 | 0.6 |
| AIDS Care | 48 | 1.2 | 6 | 1.6 | 6 | 3.0 | - | - |
| AIDS Education and Prevention | 14 | 0.3 | 2 | 0.5 | 2 | 1.0 | - | - |
| AIDS Patient Care and STDs | 14 | 0.3 | 3 | 0.8 | 3 | 1.5 | - | - |
| Annals of Behavioral Medicine | 10 | 0.2 | 1 | 0.3 | 1 | 0.5 | - | - |
| BMC Geriatrics | 80 | 1.9 | 1 | 0.3 | 1 | 0.5 | - | - |
| BMC Health Services Research | 154 | 3.7 | 8 | 2.2 | 5 | 2.5 | 3 | 1.8 |
| BMC Psychiatry | 68 | 1.6 | 5 | 1.4 | 5 | 2.5 | - | - |
| BMC Public Health | 236 | 5.7 | 12 | 3.3 | 9 | 4.5 | 3 | 1.8 |
| BMJ Open | 628 | 15.2 | 32 | 8.7 | 19 | 9.5 | 13 | 7.8 |
| Contemporary Clinical Trials | 190 | 4.6 | 78 | 21.3 | 25 | 12.5 | 53 | 31.7 |
| Diabetes | 74 | 1.8 | 8 | 2.2 | 2 | 1.0 | 6 | 3.6 |
| Frontiers in Psychology | 132 | 3.2 | 8 | 2.2 | 4 | 2.0 | 4 | 2.4 |
| Frontiers in Public Health | 79 | 1.9 | 16 | 4.4 | 14 | 7.0 | 2 | 1.2 |
| Journal of Adolescent Health | 69 | 1.7 | 6 | 1.6 | 6 | 3.0 | - | - |
| Journal of the American Academy of Child and Adolescent Psychiatry | 137 | 3.3 | 36 | 9.8 | 27 | 13.5 | 9 | 5.4 |
| Journal of Autism and Development Disorders | 91 | 2.2 | 2 | 0.5 | 2 | 1.0 | - | - |
| Journal of Medical Internet Research | 385 | 9.3 | 13 | 3.5 | 11 | 5.5 | 2 | 1.2 |
| Mindfulness | 70 | 1.7 | 1 | 0.3 | 1 | 0.5 | - | - |
| Pediatrics | 102 | 2.5 | 17 | 4.6 | 15 | 7.5 | 2 | 1.2 |
| Pilot and Feasibility Studies | 501 | 12.1 | 61 | 16.6 | 16 | 8.0 | 45 | 26.9 |
| PLOS One | 365 | 8.8 | 12 | 3.3 | 6 | 3.0 | 6 | 3.6 |
| Translational Behavior Medicine | 84 | 2.0 | 15 | 4.1 | 6 | 3.0 | 9 | 5.4 |
| Trials | 443 | 10.7 | 12 | 3.3 | 4 | 2.0 | 8 | 4.8 |

**Supplementary Table 1.** List of top 25 included journals and their citation count in the full sample, the subsample, Non-Citers, and Citers

**Supplementary Table 2.** Full list of guidelines, checklists, frameworks, and recommendations by topic with citation counts for the full sample and those in the Citers category.

| **GCFR Topic** | **Full Sample** (n=1,316) | | **Citers** (n=167) | |
| --- | --- | --- | --- | --- |
|  | **Citation Count** | **Percent (%)** | **Citation Count** | **Percent (%)** |
| **Adaptations** | 27 | 2.1 | 15 | 8.9 |
| Barrera 2006 | 3 | 0.2 | 1 | 0.6 |
| Castro 2004 | 6 | 0.5 | 2 | 1.2 |
| Chambers 2016 | 4 | 0.3 | 3 | 1.8 |
| Miller 2021 | 0 | 0.0 | 0 | 0.0 |
| Stirman 2013, 2019 | 14 | 1.1 | 9 | 5.4 |
| **Defining Pilot and Feasibility Studies** | 142 | 10.8 | 76 | 45.5 |
| Arain 2010 | 31 | 2.4 | 14 | 8.4 |
| Eldridge 2016 (Defining) | 39 | 2.9 | 18 | 10.8 |
| NIHR Definition of PFS | 0 | 0.0 | 0 | 0.0 |
| Shanyinde 2011 | 6 | 0.5 | 5 | 2.9 |
| Thabane 2010, 2017, 2019 | 62 | 4.6 | 37 | 22.2 |
| van Teijlingen 2002 | 4 | 0.1 | 2 | 1.2 |
| **Design and Interpretation** | 269 | 20.4 | 129 | 77.2 |
| Arnold 2009 | 2 | 0.2 | 0 | 0.0 |
| Bell 2018 | 7 | 0.5 | 2 | 1.2 |
| Bugge 2013 | 16 | 1.2 | 7 | 4.2 |
| Craig 2008, 2013 | 128 | 9.7 | 65 | 38.9 |
| Fletcher 2016 | 2 | 0.2 | 2 | 1.2 |
| Freedland 2020 | 0 | 0.0 | 0 | 0.0 |
| Lancaster 2004 | 56 | 4.3 | 33 | 19.8 |
| Leon 2011 | 43 | 3.3 | 10 | 5.9 |
| Levati 2016 | 4 | 0.3 | 2 | 1.2 |
| Loudon 2015 | 5 | 0.4 | 4 | 2.4 |
| MRC 2000 | 6 | 0.5 | 4 | 2.4 |
| Pearson 2020 | 0 | 0.0 | 0 | 0.0 |
| Smith 2009 | 0 | 0.0 | 0 | 0.0 |
| Westlund 2016 | 0 | 0.0 | 0 | 0.0 |
| **Feasibility** | 97 | 7.3 | 50 | 29.9 |
| Bowen 2009 | 63 | 4.8 | 27 | 16.2 |
| Huang 2018 | 2 | 0.2 | 1 | 0.6 |
| O’Cathain 2015 | 16 | 1.2 | 12 | 7.2 |
| Orsmond 2015 | 7 | 0.5 | 4 | 2.4 |
| Tickle-Degnen 2013 | 9 | 0.7 | 6 | 3.6 |
| **Guidance Review** | 22 | 1.6 | 1 | 0.6 |
| Hallingberg 2018 | 2 | 0.2 | 1 | 0.6 |
| McCrabb 2020 | 0 | 0.0 | 0 | 0.0 |
| **Implementation** | 123 | 9.3 | 50 | 29.9 |
| Braganza 2021 | 3 | 0.2 | 1 | 0.6 |
| Curran 2012 | 10 | 0.8 | 2 | 1.2 |
| Damschroder 2009 | 22 | 1.7 | 10 | 5.9 |
| Meyers 2012 | 3 | 0.2 | 1 | 0.6 |
| Pfadenhauer 2017 | 2 | 0.2 | 2 | 1.2 |
| Proctor 2011 | 30 | 2.3 | 11 | 6.6 |
| RE-AIM | 50 | 9.8 | 21 | 12.6 |
| Wandersman 2008 | 3 | 0.2 | 2 | 1.2 |
| **Intervention Development** | 35 | 2.7 | 17 | 10.2 |
| Bartholomew 1998 | 8 | 0.6 | 4 | 2.4 |
| Collin 2005 | 3 | 0.2 | 2 | 1.2 |
| French 2012 | 6 | 0.5 | 4 | 2.4 |
| Hawkins 2017 | 3 | 0.2 | 0 | 0.0 |
| Hoddinott 2015 | 5 | 0.4 | 3 | 1.8 |
| Onken 2014 | 10 | 0.8 | 4 | 2.4 |
| **Progression Criteria** | 16 | 1.2 | 9 | 5.4 |
| Avery 2017 | 16 | 1.2 | 9 | 5.4 |
| Hampson 2018 | 0 | 0.0 | 0 | 0.0 |
| **Reporting** | 281 | 21.4 | 113 | 67.7 |
| Albrecht 2013 | 0 | 0.0 | 0 | 0.0 |
| Borek 2015 | 4 | 0.3 | 4 | 2.4 |
| Chan 2013 | 32 | 2.4 | 12 | 7.2 |
| Davidson 2003 | 1 | 0.1 | 0 | 0.0 |
| Des Jarlais 2004 | 1 | 0.1 | 0 | 0.0 |
| Eldridge 2016 (CONSORT Ext.) | 123 | 9.3 | 35 | 20.9 |
| Hoffmann 2014 | 79 | 6.0 | 43 | 25.7 |
| Lancaster 2019 | 4 | 0.3 | 3 | 1.8 |
| Michie 2009 | 3 | 0.2 | 1 | 0.6 |
| Mohler 2013, 2015 | 1 | 0.1 | 0 | 0.0 |
| O’Brien 2014 | 3 | 0.2 | 2 | 1.2 |
| Ogrinc 2015 | 2 | 0.2 | 1 | 0.6 |
| Tate 2016 | 2 | 0.2 | 0 | 0.0 |
| Thabane 2019 | 2 | 0.2 | 1 | 0.6 |
| Tong 2007, 2012 | 24 | 1.8 | 11 | 6.6 |
| **Sample Size/ Power** | 76 | 5.8 | 31 | 18.6 |
| Billingham 2013 | 32 | 2.4 | 18 | 10.8 |
| Eldridge 2016 (How Big) | 1 | 0.1 | 1 | 0.6 |
| Kraemer 2006 | 17 | 1.3 | 1 | 0.6 |
| Teare 2014 | 26 | 1.9 | 11 | 6.6 |
| **Scale-Up** | 30 | 2.3 | 13 | 7.8 |
| Barker 2016 | 0 | 0.0 | 0 | 0.0 |
| Charlesworth 2013 | 4 | 0.3 | 2 | 1.2 |
| Czajkowski 2015 | 15 | 1.1 | 6 | 3.6 |
| Indig 2017 | 0 | 0.0 | 0 | 0.0 |
| Kumar 2019 | 0 | 0.0 | 0 | 0.0 |
| McCrabb 2019 | 1 | 0.1 | 0 | 0.0 |
| Milat 2011, 2013, 2015, 2016 | 2 | 0.2 | 1 | 0.6 |
| O’Hara 2014 | 0 | 0.0 | 0 | 0.0 |
| Paina 2012 | 0 | 0.0 | 0 | 0.0 |
| Rounsaville 2001 | 7 | 0.5 | 4 | 2.4 |
| Spicer 2014 | 0 | 0.0 | 0 | 0.0 |
| Subramanian 2011 | 0 | 0.0 | 0 | 0.0 |
| WHO 2010 | 1 | 0.1 | 0 | 0.0 |
| Yamey 2011 | 0 | 0.0 | 0 | 0.0 |

**Supplementary Table 3.** Operational definitions of trial- and intervention-related feasibility indicators and keywords used to search for them via text-mining.

| **Category** | **Indicator** | **Definition** | **Search Terms** |
| --- | --- | --- | --- |
| Trial-Related Feasibility Indicators | Recruitment Capability | The proportion of eligible participants who are enrolled at baseline of the study. | Search was completed manually. Information typically provided in CONSORT diagrams. |
|  | Retention | The proportion of enrolled participants who are present throughout the full length of the treatment. | Search was completed manually. Information typically provided in CONSORT diagrams. |
| Intervention-Related Feasibility Indicators | Treatment Fidelity^3^ | Content, frequency, duration, and coverage as originally intended | adhere*, deliver*, fidelity |
|  | Acceptability | The perception among participants’ that the treatment is agreeable or satisfactory. | accept*, appropriate*, enjoy*, satisf*, fun, safety, difficult*, tolera* |
|  | Adverse Events | Participant harms related to intervention procedures | adverse event*, harm*, safe*, injur*, hurt |
|  | Attendance^3^ | The proportion of total sessions offered to participants to the actual number of sessions participants attended. | attend*, engag*, |
|  | Compliance^3^ | Participants’ level of adherence to the content, frequency, duration, and coverage of the treatment as delivered by the research team. | comply, complian*, adhere* |
|  | Cost | Monetary costs associated with  delivering the intervention to  participants. | cost*, economic eval*, $, AUD, CAD,  CHF, £, pound sterling, €, euro, ¥, yen |
|  | Data Collection | Feasibility of data collection procedures | data collection, data management, collect*, measure* |

**Search Strategy for Pilot Studies**

((((("pilot"[Title/Abstract]) OR (feasibility[Title/Abstract])) OR (preliminary[Title/Abstract])) OR (proof-of-concept[Title/Abstract])) OR (vanguard[Title/Abstract])) AND (intervention[Title/Abstract])

**Reference list of guidelines, checklists, frameworks, and recommendations by topic**

***Adaptations***

Barrera M, Castro FG. A heuristic framework for the cultural adaptation of interventions. Clin Psychol-Sci Pr. 2006;13(4):311-316.

Castro FG, Barrera M, Martinez CR. The cultural adaptation of prevention interventions: Resolving tensions between fidelity and fit. Prev Sci. 2004;5(1):41-45.

Chambers DA, Norton WE. The Adaptome Advancing the Science of Intervention Adaptation. Am J Prev Med. 2016;51(4):S124-S131.

Kirk MA, Moore JE, Stirman SW, Birken SA. Towards a comprehensive model for understanding adaptations' impact: the model for adaptation design and impact (MADI). Implement Sci. 2020;15(1).

Miller CJ, Barnett ML, Baumann AA, Gutner CA, Wiltsey-Stirman S. The FRAME-IS: a framework for documenting modifications to implementation strategies in healthcare. Implement Sci. 2021;16(1).

Stirman SW, Baumann AA, Miller CJ. The FRAME: an expanded framework for reporting adaptations and modifications to evidence-based interventions. Implement Sci. 2019;14.

Stirman SW, Miller CJ, Toder K, Calloway A. Development of a framework and coding system for modifications and adaptations of evidence-based interventions. Implement Sci. 2013;8.

***Defining Pilot and Feasibility Studies***

Arain M, Campbell MJ, Cooper CL, Lancaster GA. What is a pilot or feasibility study? A review of current practice and editorial policy. Bmc Medical Research Methodology. 2010;10.

Eldridge SM, Lancaster GA, Campbell MJ, et al. Defining Feasibility and Pilot Studies in Preparation for Randomised Controlled Trials: Development of a Conceptual Framework. Plos One. 2016;11(3).

Shanyinde M, Pickering RM, Weatherall M. Questions asked and answered in pilot and feasibility randomized controlled trials. Bmc Medical Research Methodology. 2011;11.

Thabane L, Cambon L, Potvin L, et al. Population health intervention research: what is the place for pilot studies? Trials. 2019;20(1):309.

Thabane L, Ma J, Chu R, et al. A tutorial on pilot studies: the what, why and how. Bmc Medical Research Methodology. 2010;10.

van Teijlingen E, Hundley V. The importance of pilot studies. Nurs Stand. 2002;16(40):33-36.

***Design and Interpretation***

Arnold DM, Burns KEA, Adhikari NKJ, et al. The design and interpretation of pilot trials in clinical research in critical care. Critical Care Medicine. 2009;37(1):S69-S74.

Bell ML, Whitehead AL, Julious SA. Guidance for using pilot studies to inform the design of intervention trials with continuous outcomes. Clin Epidemiol. 2018;10:153-157.

Bugge C, Williams B, Hagen S, et al. A process for Decision-making after Pilot and feasibility Trials (ADePT): development following a feasibility study of a complex intervention for pelvic organ prolapse. Trials. 2013;14.

Craig P, Dieppe P, Macintyre S, Michie S, Nazareth I, Petticrew M. Developing and evaluating complex interventions: the new Medical Research Council guidance. Brit Med J. 2008;337(7676).

Craig P, Dieppe P, Macintyre S, Michie S, Nazareth I, Petticrew M. Developing and evaluating complex interventions: The new Medical Research Council guidance. Int J Nurs Stud. 2013;50(5):587-592.

Fletcher A, Jamal F, Moore G, Evans RE, Murphy S, Bonell C. Realist complex intervention science: Applying realist principles across all phases of the Medical Research Council framework for developing and evaluating complex interventions. Evaluation-Us. 2016;22(3):286-303.

Freedland KE. Pilot Trials in Health-Related Behavioral Intervention Research: Problems, Solutions, and Recommendations. Health Psychology. 2020;39(10):851-862.

Lancaster GA, Dodd S, Williamson PR. Design and analysis of pilot studies: recommendations for good practice. J Eval Clin Pract. 2004;10(2):307-312.

Leon AC, Davis LL, Kraemer HC. The role and interpretation of pilot studies in clinical research. J Psychiatr Res. 2011;45(5):626-629.

Levati S, Campbell P, Frost R, et al. Optimisation of complex health interventions prior to a randomised controlled trial: a scoping review of strategies used. Pilot Feasibility Stud. 2016;2:17.

Loudon K, Treweek S, Sullivan F, Donnan P, Thorpe KE, Zwarenstein M. The PRECIS-2 tool: designing trials that are fit for purpose. Bmj-Brit Med J. 2015;350.

Pearson N, Naylor PJ, Ashe MC, Fernandez M, Yoong SL, Wolfenden L. Guidance for conducting feasibility and pilot studies for implementation trials. Pilot and Feasibility Studies. 2020;6(1).

Smith LJ, Harrison MB. Framework for Planning and Conducting Pilot Studies. Ostomy Wound Manag. 2009;55(12):34-+.

Westlund E, Stuart EA. The Nonuse, Misuse, and Proper Use of Pilot Studies in Experimental Evaluation Research. Am J Eval. 2017;38(2):246-261.

***Feasibility***

Bowen DJ, Kreuter M, Spring B, et al. How We Design Feasibility Studies. Am J Prev Med. 2009;36(5):452-457.

Huang GD, Bull J, McKee KJ, et al. Clinical trials recruitment planning: A proposed framework from the Clinical Trials Transformation Initiative. Contemporary Clinical Trials. 2018;66:74-79.

O'Cathain A, Hoddinott P, Lewin S, et al. Maximising the impact of qualitative research in feasibility studies for randomised controlled trials: guidance for researchers. Pilot Feasibility Stud. 2015;1:32.

Orsmond GI, Cohn ES. The Distinctive Features of a Feasibility Study: Objectives and Guiding Questions. Otjr-Occup Part Heal. 2015;35(3):169-177.

Tickle-Degnen L. Nuts and Bolts of Conducting Feasibility Studies. Am J Occup Ther. 2013;67(2):171-176.

***Guidance Review***

Hallingberg B, Turley R, Segrott J, et al. Exploratory studies to decide whether and how to proceed with full-scale evaluations of public health interventions: a systematic review of guidance. Pilot Feasibility Stud. 2018;4:104.

McCrabb S, Mooney K, Elton B, Grady A, Yoong SL, Wolfenden L. How to optimise public health interventions: a scoping review of guidance from optimisation process frameworks. Bmc Public Health. 2020;20(1).

***Implementation***

Braganza MZ, Kilbourne AM. The Quality Enhancement Research Initiative (QUERI) Impact Framework: Measuring the Real-world Impact of Implementation Science. J Gen Intern Med. 2021;36(2):396-403.

Curran GM, Bauer M, Mittman B, Pyne JM, Stetler C. Effectiveness-implementation Hybrid Designs Combining Elements of Clinical Effectiveness and Implementation Research to Enhance Public Health Impact. Med Care. 2012;50(3):217-226.

Damschroder LJ, Aron DC, Keith RE, Kirsh SR, Alexander JA, Lowery JC. Fostering implementation of health services research findings into practice: a consolidated framework for advancing implementation science. Implement Sci. 2009;4.

Glasgow RE, Vogt TM, Boles SM. Evaluating the public health impact of health promotion interventions: the RE-AIM framework. Am J Public Health. 1999;89(9):1322-1327.

Meyers DC, Durlak JA, Wandersman A. The Quality Implementation Framework: A Synthesis of Critical Steps in the Implementation Process. Am J Commun Psychol. 2012;50(3-4):462-480.

Pfadenhauer LM, Gerhardus A, Mozygemba K, et al. Making sense of complexity in context and implementation: the Context and Implementation of Complex Interventions (CICI) framework. Implement Sci. 2017;12.

Proctor E, Silmere H, Raghavan R, et al. Outcomes for Implementation Research: Conceptual Distinctions, Measurement Challenges, and Research Agenda. Adm Policy Ment Hlth. 2011;38(2):65-76.

Wandersman A, Duffy J, Flaspohler P, et al. Bridging the gap between prevention research and practice: The interactive systems framework for dissemination and implementation. Am J Commun Psychol. 2008;41(3-4):171-181.

***Intervention Development***

Bartholomew LK, Parcel GS, Kok G. Intervention mapping: A process for developing theory- and evidence-based health education programs. Health Educ Behav. 1998;25(5):545-563.

Collins LM, Murphy SA, Nair VN, Strecher VJ. A strategy for optimizing and evaluating behavioral interventions. Annals of Behavioral Medicine. 2005;30(1):65-73.

French SD, Green SE, O'Connor DA, et al. Developing theory-informed behaviour change interventions to implement evidence into practice: a systematic approach using the Theoretical Domains Framework. Implement Sci. 2012;7.

Hawkins J, Madden K, Fletcher A, et al. Development of a framework for the co-production and prototyping of public health interventions. Bmc Public Health. 2017;17.

Hoddinott P. A new era for intervention development studies. Pilot Feasibility Stud. 2015;1:36.

Onken LS, Carroll KM, Shoham V, Cuthbert BN, Riddle M. Reenvisioning Clinical Science: Unifying the Discipline to Improve the Public Health. Clin Psychol Sci. 2014;2(1):22-34.

***Progression Criteria***

Avery KNL, Williamson PR, Gamble C, et al. Informing efficient randomised controlled trials: exploration of challenges in developing progression criteria for internal pilot studies. Bmj Open. 2017;7(2).

Hampson LV, Williamson PR, Wilby MJ, Jaki T. A framework for prospectively defining progression rules for internal pilot studies monitoring recruitment. Stat Methods Med Res. 2018;27(12):3612-3627.

***Reporting***

Albrecht L, Archibald M, Arseneau D, Scott SD. Development of a checklist to assess the quality of reporting of knowledge translation interventions using the Workgroup for Intervention Development and Evaluation Research (WIDER) recommendations. Implement Sci. 2013;8.

Borek AJ, Abraham C, Smith JR, Greaves CJ, Tarrant M. A checklist to improve reporting of group-based behaviour-change interventions. Bmc Public Health. 2015;15.

Chan AW, Tetzlaff JM, Gotzsche PC, et al. SPIRIT 2013 explanation and elaboration: guidance for protocols of clinical trials. BMJ. 2013;346:e7586.

Davidson KW, Goldstein M, Kaplan RM, et al. Evidence-based behavioral medicine: What is it and how do we achieve it? Annals of Behavioral Medicine. 2003;26(3):161-171.

Des Jarlais DC, Lyles C, Crepaz N, Grp T. Improving the reporting quality of nonrandomized evaluations of behavioral and public health interventions: The TREND statement. Am J Public Health. 2004;94(3):361-366.

Eldridge SM, Chan CL, Campbell MJ, et al. CONSORT 2010 statement: extension to randomised pilot and feasibility trials. Pilot Feasibility Stud. 2016;2:64.

Hoffmann TC, Glasziou PP, Boutron I, et al. Better reporting of interventions: template for intervention description and replication (TIDieR) checklist and guide. BMJ. 2014;348:g1687.

Lancaster GA, Thabane L. Guidelines for reporting non-randomised pilot and feasibility studies. Pilot and Feasibility Studies. 2019;5(1).

Michie S, Fixsen D, Grimshaw JM, Eccles MP. Specifying and reporting complex behaviour change interventions: the need for a scientific method. Implement Sci. 2009;4.

Mohler R, Bartoszek G, Meyer G. Quality of reporting of complex healthcare interventions and applicability of the CReDECI list - a survey of publications indexed in PubMed. Bmc Medical Research Methodology. 2013;13.

Mohler R, Kopke S, Meyer G. Criteria for Reporting the Development and Evaluation of Complex Interventions in healthcare: revised guideline (CReDECI 2). Trials. 2015;16.

O'Brien BC, Harris IB, Beckman TJ, Reed DA, Cook DA. Standards for reporting qualitative research: a synthesis of recommendations. Acad Med. 2014;89(9):1245-1251.

Ogrinc G, Davies L, Goodman D, Batalden P, Davidoff F, Stevens D. SQUIRE 2.0 (Standards for QUality Improvement Reporting Excellence): Revised Publication Guidelines From a Detailed Consensus Process. J Contin Educ Nurs. 2015;46(11):501-507.

Thabane L, Lancaster G. A guide to the reporting of protocols of pilot and feasibility trials. Pilot and Feasibility Studies. 2019;5(1).

Tong A, Flemming K, McInnes E, Oliver S, Craig J. Enhancing transparency in reporting the synthesis of qualitative research: ENTREQ. BMC Med Res Methodol. 2012;12:181.

Tong A, Sainsbury P, Craig J. Consolidated criteria for reporting qualitative research (COREQ): a 32-item checklist for interviews and focus groups. Int J Qual Health Care. 2007;19(6):349-357.

***Sample Size/Power***

Billingham SAM, Whitehead AL, Julious SA. An audit of sample sizes for pilot and feasibility trials being undertaken in the United Kingdom registered in the United Kingdom Clinical Research Network database. Bmc Medical Research Methodology. 2013;13.

Eldridge SM, Costelloe CE, Kahan BC, Lancaster GA, Kerry SM. How big should the pilot study for my cluster randomised trial be? Statistical Methods in Medical Research. 2016;25(3):1039-1056.

Kraemer HC, Mintz J, Noda A, Tinklenberg J, Yesavage JA. Caution regarding the use of pilot studies to guide power calculations for study proposals. Arch Gen Psychiat. 2006;63(5):484-489.

Teare MD, Dimairo M, Shephard N, Hayman A, Whitehead A, Walters SJ. Sample size requirements to estimate key design parameters from external pilot randomised controlled trials: a simulation study. Trials. 2014;15.

***Scale-Up***

Baier RR, Jutkowitz E, Mitchell SL, McCreedy E, Mor V. Readiness assessment for pragmatic trials (RAPT): a model to assess the readiness of an intervention for testing in a pragmatic trial. BMC Med Res Methodol. 2019;19(1):156.

Barker PM, Reid A, Schall MW. A framework for scaling up health interventions: lessons from large-scale improvement initiatives in Africa. Implement Sci. 2016;11.

Charlesworth G, Burnell K, Hoe J, Orrell M, Russell I. Acceptance checklist for clinical effectiveness pilot trials: a systematic approach. BMC Med Res Methodol. 2013;13:78.

Czajkowski SM, Powell LH, Adler N, et al. From Ideas to Efficacy: The ORBIT Model for Developing Behavioral Treatments for Chronic Diseases. Health Psychology. 2015;34(10):971-982.

Indig D, Lee K, Grunseit A, Milat A, Bauman A. Pathways for scaling up public health interventions. Bmc Public Health. 2017;18.

Kumar S, Dave P, Srivastava A, et al. Harmonizing scientific rigor with political urgency: policy learnings for identifying accelerators for scale-up from the safe childbirth checklist programme in Rajasthan, India. Bmc Health Services Research. 2019;19.

McCrabb S, Lane C, Hall A, et al. Scaling-up evidence-based obesity interventions: A systematic review assessing intervention adaptations and effectiveness and quantifying the scale-up penalty. Obes Rev. 2019;20(7):964-982.

Milat AJ, Bauman A, Redman S. Narrative review of models and success factors for scaling up public health interventions. Implement Sci. 2015;10.

Milat AJ, King L, Bauman A, Redman S. Scaling up health promotion interventions: an emerging concept in implementation science. Health Promot J Austr. 2011;22(3):238.

Milat AJ, King L, Bauman AE, Redman S. The concept of scalability: increasing the scale and potential adoption of health promotion interventions into policy and practice. Health Promot Int. 2013;28(3):285-298.

Milat AJ, Newson R, King L, et al. A guide to scaling up population health interventions. Public Health Res Pract. 2016;26(1):e2611604.

Mummah SA, Robinson TN, King AC, Gardner CD, Sutton S. IDEAS (Integrate, Design, Assess, and Share): A Framework and Toolkit of Strategies for the Development of More Effective Digital Interventions to Change Health Behavior. J Med Internet Res. 2016;18(12):e317.

O'Hara BJ, Phongsavan P, King L, et al. 'Translational formative evaluation': critical in up-scaling public health programmes. Health Promot Int. 2014;29(1):38-46.

Paina L, Peters DH. Understanding pathways for scaling up health services through the lens of complex adaptive systems. Health Policy Plann. 2012;27(5):365-373.

Spicer N, Bhattacharya D, Dimka R, et al. 'Scaling-up is a craft not a science': Catalysing scale-up of health innovations in Ethiopia, India and Nigeria. Soc Sci Med. 2014;121:30-38.

Subramanian S, Naimoli J, Matsubayashi T, Peters DH. Do we have the right models for scaling up health services to achieve the Millennium Development Goals? Bmc Health Services Research. 2011;11.

World Health Organization. Nine steps for developing a scaling-up strategy. 2010.

Yamey G. Scaling Up Global Health Interventions: A Proposed Framework for Success. Plos Med. 2011;8(6).

**Reference list of included studies**

1.Abas M, Nyamayaro P, Bere T, et al. Feasibility and Acceptability of a Task-Shifted Intervention to Enhance Adherence to HIV Medication and Improve Depression in People Living with HIV in Zimbabwe, a Low Income Country in Sub-Saharan Africa. Aids and Behavior. 2018;22(1):86-101.

2.Abdel-All M, Thrift AG, Riddell M, et al. Evaluation of a training program of hypertension for accredited social health activists (ASHA) in rural India. BMC Health Serv Res. 2018;18(1):320.

3.Adinan J, Adamou B, Amour C, Shayo A, Kidayi PL, Msuya L. Feasibility of home-based HIV counselling and testing and linking to HIV services among women delivering at home in Geita, Tanzania: a household longitudinal survey. Bmc Public Health. 2019;19(1).

4.Agarwal P, Kithulegoda N, Bouck Z, et al. Feasibility of an Electronic Health Tool to Promote Physical Activity in Primary Care: Pilot Cluster Randomized Controlled Trial. J Med Internet Res. 2020;22(2):e15424.

5.Ahmed S, Dupuis V, Tyron M, et al. Intended and Unintended Consequences of a Community-Based Fresh Fruit and Vegetable Dietary Intervention on the Flathead Reservation of the Confederated Salish and Kootenai Tribes. Front Public Health. 2020;8:331.

6.Aji M, Glozier N, Bartlett D, et al. A feasibility study of a mobile app to treat insomnia. Transl Behav Med. 2021;11(2):604-612.

7.Algotar AM, Kumar R, Babiker HM, et al. Protocol for a feasibility and early efficacy study of the Comprehensive Lifestyle Improvement Program for Prostate Cancer-2 (CLIPP2). Contemp Clin Trials Commun. 2021;21:100701.

8.Allan LM. Is it feasible to deliver a complex intervention to improve the outcome of falls in people with dementia? A protocol for the DIFRID feasibility study. Pilot Feasibility Stud. 2018.

9.Almas A. School health education program in Pakistan (SHEPP)-a threefold health education feasibility trial in schoolchildren from a lower-middle-income country. Pilot Feasibility Stud. 2020.

10.Alon D, Sousa CV, Baranowski T, et al. The impact of narratives and active video games on long-term moderate-to-vigorous physical activity: A randomized controlled trial protocol. Contemp Clin Trials. 2020;96:106087.

11.Alrushud AS, Rushton AB, Bhogal G, Pressdee F, Greig CA. Effect of a combined programme of dietary restriction and physical activity on the physical function and body composition of obese middle-aged and older adults with knee OA (DRPA): protocol for a feasibility study. BMJ Open. 2018;8(12):e021051.

12.Alsubaie M, Dickens C, Dunn BD, et al. Feasibility and Acceptability of Mindfulness-based Cognitive Therapy Compared with Mindfulness-based Stress Reduction and Treatment as Usual in People with Depression and Cardiovascular Disorders: a Three-Arm Randomised Controlled Trial. Mindfulness. 2020;11(1):30-50.

13.Alzahrani H, Mackey M, Stamatakis E, Shirley D. Wearables-based walking program in addition to usual physiotherapy care for the management of patients with low back pain at medium or high risk of chronicity: A pilot randomized controlled trial. Plos One. 2021;16(8).

14.Aminian S, Motl RW, Rowley J, Manns PJ. Management of multiple sclerosis symptoms through reductions in sedentary behaviour: protocol for a feasibility study. Bmj Open. 2019;9(4).

15.Andermo S, Hellenius ML, Lidin M, Hedby U, Nordenfelt A, Nyberg G. Effectiveness of a family intervention on health-related quality of life-a healthy generation, a controlled pilot trial. Bmc Public Health. 2020;20(1).

16.Anderson AS, Craigie AM, Gallant S, et al. Optimisation of the ActWELL lifestyle intervention programme for women attending routine NHS breast screening clinics. Trials. 2020;21(1):484.

17.Arikawa AY, Kaufman BC, Raatz SK, Kurzer MS. Effects of a parallel-arm randomized controlled weight loss pilot study on biological and psychosocial parameters of overweight and obese breast cancer survivors. Pilot Feasibility Stud. 2018;4:17.

18.Aschbrenner KA, Patten CA, Brunette MF. Feasibility of a support person intervention to promote smoking cessation treatment use among smokers with mental illness. Translational Behavioral Medicine. 2018;8(5):785-792.

19.Asrat B, Lund C, Ambaw F, Schneider M. Acceptability and feasibility of peer-administered group interpersonal therapy for depression for people living with HIV/AIDS-a pilot study in Northwest Ethiopia. Pilot Feasibility Stud. 2021;7(1):147.

20.Audsley S, Kendrick D, Logan P, Jones M, Orton E. A randomised feasibility study assessing an intervention to keep adults physically active after falls management exercise programmes end. Pilot Feasibility Stud. 2020;6:37.

21.Baggett TP, Yaqubi A, Berkowitz SA, et al. Subsistence difficulties are associated with more barriers to quitting and worse abstinence outcomes among homeless smokers: evidence from two studies in Boston, Massachusetts. Bmc Public Health. 2018;18.

22.Bajwa RK, Goldberg SE, Van der Wardt V, et al. A randomised controlled trial of an exercise intervention promoting activity, independence and stability in older adults with mild cognitive impairment and early dementia (PrAISED) - A Protocol. Trials. 2019;20(1).

23.Bana M, Ribi K, Kropf-Staub S, et al. Implementation of the Symptom Navi (c) Programme for cancer patients in the Swiss outpatient setting: a study protocol for a cluster randomised pilot study (Symptom Navi(c) Pilot Study). BMJ Open. 2019;9(7):e027942.

24.Barnett M, Miranda J, Kia-Keating M, Saldana L, Landsverk J, Lau AS. Developing and evaluating a lay health worker delivered implementation intervention to decrease engagement disparities in behavioural parent training: a mixed methods study protocol. BMJ Open. 2019;9(7):e028988.

25.Baskerville NB, Struik LL, Dash D. Crush the Crave: Development and Formative Evaluation of a Smartphone App for Smoking Cessation. JMIR Mhealth Uhealth. 2018;6(3):e52.

26.Bassett SM, Brody LR, Jack DC, et al. Feasibility and Acceptability of a Program to Promote Positive Affect, Well-Being and Gender Empowerment in Black Women Living with HIV. Aids and Behavior. 2021;25(6):1737-1750.

27.Beck KB, Greco CM, Terhorst LA, Skidmore ER, Kulzer JL, McCue MP. Mindfulness-Based Stress Reduction for Adults with Autism Spectrum Disorder: Feasibility and Estimated Effects. Mindfulness. 2020;11(5):1286-1297.

28.Bell LV, Cornish P, Flusk D, Garland SN, Rash JA. The INternet ThERapy for deprESsion Trial (INTEREST): protocol for a patient-preference, randomised controlled feasibility trial comparing iACT, iCBT and attention control among individuals with comorbid chronic pain and depression. Bmj Open. 2020;10(2).

29.Bellone KM, Elliott SC, Hynan LS, Warren B, Jarrett RB. Mindful Self-Care for Caregivers: A Proof of Concept Study Investigating a Model for Embedded Caregiver Support in a Pediatric Setting. J Autism Dev Disord. 2021.

30.Bendig E, Bauereiss N, Schmitt A, Albus P, Baumeister H. ACTonDiabetes-a guided psychological internet intervention based on Acceptance and Commitment Therapy (ACT) for adults living with type 1 or 2 diabetes: results of a randomised controlled feasibility trial. BMJ Open. 2021;11(7):e049238.

31.Bentley CL, Powell L, Potter S, et al. The Use of a Smartphone App and an Activity Tracker to Promote Physical Activity in the Management of Chronic Obstructive Pulmonary Disease: Randomized Controlled Feasibility Study. JMIR Mhealth Uhealth. 2020;8(6):e16203.

32.Bergstrom A, Borell L, Meijer S, Guidetti S. Evaluation of an intervention addressing a reablement programme for older, community-dwelling persons in Sweden (ASSIST 1.0): a protocol for a feasibility study. Bmj Open. 2019;9(7).

33.Berkel C, Smith JD, Bruening MM, et al. The Family Check-Up 4 Health: Study protocol of a randomized type II hybrid effectiveness-implementation trial in integrated primary care (the healthy communities 4 healthy students study). Contemp Clin Trials. 2020;96:106088.

34.Betthauser LM, Forster JE, Bortz A, et al. Strength and awareness in action: Feasibility of a yoga-based intervention for post-acute mild TBI headaches among veterans. Contemp Clin Trials Commun. 2021;22:100762.

35.Bierhoff M, Nelson KE, Guo N, et al. Prevention of mother-to-child transmission of hepatitis B virus: protocol for a one-arm, open-label intervention study to estimate the optimal timing of tenofovir in pregnancy. Bmj Open. 2020;10(9).

36.Billany RE. "A pilot randomised controlled trial of a structured, home-based exercise programme on cardiovascular structure and function in kidney transplant recipients: the ECSERT study design and methods". BMJ Open. 2021.

37.Bjureberg J, Sahlin H, Hedman-Lagerlof E, et al. Extending research on Emotion Regulation Individual Therapy for Adolescents (ERITA) with nonsuicidal self-injury disorder: open pilot trial and mediation analysis of a novel online version. BMC Psychiatry. 2018;18(1):326.

38.Borek AJ, McDonald B, Fredlund M, Bjornstad G, Logan S, Morris C. Healthy Parent Carers programme: development and feasibility of a novel group-based health-promotion intervention. BMC Public Health. 2018;18(1):270.

39.Borosund E, Varsi C, Clark MM, et al. Pilot testing an app-based stress management intervention for cancer survivors. Transl Behav Med. 2020;10(3):770-780.

40.Bowden JL, Egerton T, Hinman RS, et al. Protocol for the process and feasibility evaluations of a new model of primary care service delivery for managing pain and function in patients with knee osteoarthritis (PARTNER) using a mixed methods approach. Bmj Open. 2020;10(2).

41.Brady B, Veljanova I, Schabrun S, Chipchase L. Integrating culturally informed approaches into physiotherapy assessment and treatment of chronic pain: a pilot randomised controlled trial. BMJ Open. 2018;8(7):e021999.

42.Braun T, Gruneberg C, Sussmilch K, et al. An augmented prescribed exercise program (APEP) to improve mobility of older acute medical patients - a randomized, controlled pilot and feasibility trial. Bmc Geriatrics. 2019;19(1).

43.Brewer SE, Cataldi JR, Fisher M, Glasgow RE, Garrett K, O'Leary ST. Motivational Interviewing for Maternal Immunisation (MI4MI) study: a protocol for an implementation study of a clinician vaccine communication intervention for prenatal care settings. BMJ Open. 2020;10(11):e040226.

44.Broder-Fingert S, Kuhn J, Sheldrick RC, et al. Using the Multiphase Optimization Strategy (MOST) framework to test intervention delivery strategies: a study protocol. Trials. 2019;20(1).

45.Brodhead MT, Kim SY, Rispoli MJ, Sipila ES, Bak MYS. A Pilot Evaluation of a Treatment Package to Teach Social Conversation via Video-Chat. J Autism Dev Disord. 2019;49(8):3316-3327.

46.Brooker PG, Gomersall SR, King NA, Leveritt MD. The feasibility and acceptability of morning versus evening exercise for overweight and obese adults: A randomized controlled trial. Contemp Clin Trials Commun. 2019;14:100320.

47.Brown M, Murphy M, McDermott L, et al. Exercise for advanced prostate cancer: a multicomponent, feasibility, trial protocol for men with metastatic castrate-resistant prostate cancer (EXACT). Pilot and Feasibility Studies. 2019;5(1).

48.Bryant C, Brown L, Polacsek M, Batchelor F, Capon H, Dow B. Volunteer-led behavioural activation to reduce depression in residential care: a feasibility study. Pilot Feasibility Stud. 2020;6:95.

49.Bryant M, Collinson M, Burton W, et al. Cluster randomised controlled feasibility study of HENRY: a community-based intervention aimed at reducing obesity rates in preschool children. Pilot and Feasibility Studies. 2021;7(1).

50.Buchele N, Keller L, Zeller AC, et al. The effects of pre-intervention mindset induction on a brief intervention to increase risk perception and reduce alcohol use among university students: A pilot randomized controlled trial. Plos One. 2020;15(9).

51.Burrell TL, Postorino V, Scahill L, et al. Feasibility of Group Parent Training for Children with Autism Spectrum Disorder and Disruptive Behavior: A Demonstration Pilot. J Autism Dev Disord. 2020;50(11):3883-3894.

52.Cameron KL, McGinley JL, Allison K, Fini NA, Cheong JLY, Spittle AJ. Dance PREEMIE, a Dance PaRticipation intervention for Extremely prEterm children with Motor Impairment at prEschool age: an Australian feasibility trial protocol. Bmj Open. 2020;10(1).

53.Carpenter JG, Hanson LC, Hodgson N, et al. Implementing Primary Palliative Care in Post-acute nursing home care: Protocol for an embedded pilot pragmatic trial. Contemp Clin Trials Commun. 2021;23:100822.

54.Cassidy S, Okwose N, Scragg J, et al. Assessing the feasibility and acceptability of Changing Health for the management of prediabetes: protocol for a pilot study of a digital behavioural intervention. Pilot Feasibility Stud. 2019;5:139.

55.Cavallo DN, Martinez R, Webb Hooper M, Flocke S. Feasibility of a social media-based weight loss intervention designed for low-SES adults. Transl Behav Med. 2021;11(4):981-992.

56.Chandler R, Hull S, Ross H, et al. The pre-exposure prophylaxis (PrEP) consciousness of black college women and the perceived hesitancy of public health institutions to curtail HIV in black women. BMC Public Health. 2020;20(1):1172.

57.Chirico F, Sharma M, Zaffina S, Magnavita N. Spirituality and Prayer on Teacher Stress and Burnout in an Italian Cohort: A Pilot, Before-After Controlled Study. Front Psychol. 2019;10:2933.

58.Chory A, Callen G, Nyandiko W, et al. A Pilot Study of a Mobile Intervention to Support Mental Health and Adherence Among Adolescents Living with HIV in Western Kenya. Aids and Behavior. 2021.

59.Christodoulou J. Crystal Clear with Active Visualization: Understanding Medication Adherence Among Youth Living with HIV. AIDS Behav. 2020.

60.Christopher M, Bowen S, Witkiewitz K. Mindfulness-based resilience training for aggression, stress and health in law enforcement officers: study protocol for a multisite, randomized, single-blind clinical feasibility trial. Trials. 2020;21(1).

61.Chudleigh J, Holder P, Moody L, et al. Process evaluation of co-designed interventions to improve communication of positive newborn bloodspot screening results. BMJ Open. 2021;11(8):e050773.

62.Chun HY, Carson AJ, Dennis MS, Mead GE, Whiteley WN. Treating anxiety after stroke (TASK): the feasibility phase of a novel web-enabled randomised controlled trial. Pilot Feasibility Stud. 2018;4:139.

63.Clark E, Ward NS, Baio G, Jones F. Research protocol: investigating the feasibility of a group self-management intervention for stroke (the GUSTO study). Pilot Feasibility Stud. 2018;4:31.

64.Clark IN. """It's Feasible to Write a Song"": A Feasibility Study Examining Group Therapeutic Songwriting for People Living With Dementia and Their Family Caregivers". Front Psychol. 2020.

65.Clemes SA. Stand out in Class: Restructuring the classroom environment to reduce sedentary behaviour in 9-10-yearolds - Study protocol for a pilot cluster randomised controlled trial. Pilot and Feasibility Studies. 2018.

66.Collins D, Ciobanu A, Laatikainen T, et al. Protocol for the evaluation of a pilot implementation of essential interventions for the prevention of cardiovascular diseases in primary healthcare in the Republic of Moldova. BMJ Open. 2019;9(7):e025705.

67.Collins D, Inglin L, Laatikainen T, et al. Evaluation and pilot implementation of essential interventions for the management of hypertension and prevention of cardiovascular diseases in primary health care in the Republic of Tajikistan. BMC Health Serv Res. 2021;21(1):472.

68.Cordeiro C, Magalhaes S, Rocha R, et al. Promoting Third Graders' Executive Functions and Literacy: A Pilot Study Examining the Benefits of Mindfulness vs. Relaxation Training. Front Psychol. 2021;12:643794.

69.Cotter EW, Hornack SE, Fotang JP, Pettit E, Mirza NM. A pilot open-label feasibility trial examining an adjunctive mindfulness intervention for adolescents with obesity. Pilot Feasibility Stud. 2020;6:79.

70.Courtenay M, Lim R, Deslandes R, et al. Theory-based electronic learning intervention to support appropriate antibiotic prescribing by nurses and pharmacists: intervention development and feasibility study protocol. BMJ Open. 2019;9(8):e028326.

71.Crocker AM, Kessler R, van Eeghen C, et al. Integrating Behavioral Health and Primary Care (IBH-PC) to improve patient-centered outcomes in adults with multiple chronic medical and behavioral health conditions: study protocol for a pragmatic cluster-randomized control trial. Trials. 2021;22(1):200.

72.Cupertino AP, Cartujano-Barrera F, Ramirez M, et al. A Mobile Smoking Cessation Intervention for Mexico (Vive sin Tabaco... inverted exclamation markDecidete!): Single-Arm Pilot Study. JMIR Mhealth Uhealth. 2019;7(4):e12482.

73.Cvencek D, Paz-Albo J, Master A, Herranz Llacer CV, Hervas-Escobar A, Meltzoff AN. Math Is for Me: A Field Intervention to Strengthen Math Self-Concepts in Spanish-Speaking 3rd Grade Children. Front Psychol. 2020;11:593995.

74.Darvall JN, Wang A, Nazeem MN, et al. A Pedometer-Guided Physical Activity Intervention for Obese Pregnant Women (the Fit MUM Study): Randomized Feasibility Study. JMIR Mhealth Uhealth. 2020;8(5):e15112.

75.Daugherty DA, Runyan JD, Steenbergh TA, Fratzke BJ, Fry BN, Westra E. Smartphone delivery of a hope intervention: Another way to flourish. Plos One. 2018;13(6).

76.Day C, Briskman J, Crawford MJ, et al. Randomised feasibility trial of the helping families programme-modified: an intensive parenting intervention for parents affected by severe personality difficulties. BMJ Open. 2020;10(2):e033637.

77.De Panfilis L, Veronese S, Bruzzone M, et al. Study protocol on advance care planning in multiple sclerosis (ConCure-SM): intervention construction and multicentre feasibility trial. BMJ Open. 2021;11(8):e052012.

78.Deady M, Glozier N, Collins D, et al. The Utility of a Mental Health App in Apprentice Workers: A Pilot Study. Front Public Health. 2020;8:389.

79.Debenham J, Birrell L, Champion K, Askovic M, Newton N. A pilot study of a neuroscience-based, harm minimisation programme in schools and youth centres in Australia. Bmj Open. 2020;10(2).

80.Decker MR, Wood SN, Kennedy SR, et al. Adapting the myPlan safety app to respond to intimate partner violence for women in low and middle income country settings: app tailoring and randomized controlled trial protocol. BMC Public Health. 2020;20(1):808.

81.Degroote L, Van Dyck D, De Bourdeaudhuij I, De Paepe A, Crombez G. Acceptability and feasibility of the mHealth intervention 'MyDayPlan' to increase physical activity in a general adult population. Bmc Public Health. 2020;20(1).

82.Deja E, Peters MJ, Khan I, et al. Establishing and augmenting views on the acceptability of a paediatric critical care randomised controlled trial (the FEVER trial): a mixed methods study. BMJ Open. 2021;11(3):e041952.

83.Delaney MC. Caring for the caregivers: Evaluation of the effect of an eight-week pilot mindful self-compassion (MSC) training program on nurses' compassion fatigue and resilience. PLoS One. 2018;13(11):e0207261.

84.Delrieu L, Pialoux V, Perol O, et al. Feasibility and Health Benefits of an Individualized Physical Activity Intervention in Women With Metastatic Breast Cancer: Intervention Study. JMIR Mhealth Uhealth. 2020;8(1):e12306.

85.Dewitt S, Hall J, Smith L, et al. Office workers' experiences of attempts to reduce sitting-time: an exploratory, mixed-methods uncontrolled intervention pilot study. Bmc Public Health. 2019;19.

86.Dhonju G, Kunwar AR, Karki U, Devkota N, Bista I, Sah R. Identification and Management of COVID-19 Related Child and Adolescent Mental Health Problems: A Multi-Tier Intervention Model. Front Public Health. 2020;8:590002.

87.Dillingham R, Ingersoll K, Flickinger TE, et al. PositiveLinks: A Mobile Health Intervention for Retention in HIV Care and Clinical Outcomes with 12-Month Follow-Up. AIDS Patient Care STDS. 2018;32(6):241-250.

88.Doernberg EA, Russ SW, Dimitropoulos A. Believing in Make-Believe: Efficacy of a Pretend Play Intervention for School-Aged Children with High-Functioning Autism Spectrum Disorder. J Autism Dev Disord. 2021;51(2):576-588.

89.Dolcos S, Hu Y, Williams C, et al. Cultivating Affective Resilience: Proof-of-Principle Evidence of Translational Benefits From a Novel Cognitive-Emotional Training Intervention. Front Psychol. 2021;12:585536.

90.Dombrowski SU, McDonald M, van der Pol M, et al. Game of Stones: feasibility randomised controlled trial of how to engage men with obesity in text message and incentive interventions for weight loss. Bmj Open. 2020;10(2).

91.Eaton AD, Walmsley SL, Craig SL, et al. Protocol for a pilot randomised controlled trial evaluating feasibility and acceptability of cognitive remediation group therapy compared with mutual aid group therapy for people ageing with HIV-associated neurocognitive disorder (HAND) in Toronto, Canada. BMJ Open. 2019;9(10):e033183.

92.Edwards DJ, Rainey E, Boukouvala V, et al. Novel ACT-based eHealth psychoeducational intervention for students with mental distress: a study protocol for a mixed-methodology pilot trial. BMJ Open. 2019;9(7):e029411.

93.Eisenhauer CM, Brito F, Kupzyk K, et al. Mobile health assisted self-monitoring is acceptable for supporting weight loss in rural men: a pragmatic randomized controlled feasibility trial. Bmc Public Health. 2021;21(1).

94.Eisman AB, Hicks M, Kernsmith PD, Rupp L, Smith-Darden JP, Zimmerman MA. Adapting an evidence-based positive youth development intervention to prevent sexual and teen dating violence. Transl Behav Med. 2021;11(1):74-86.

95.Evans APB, Goodman SH, Dimidjian S, Gallop R. The Role of Engagement in Mindfulness-Based Cognitive Therapy for the Prevention of Depressive Relapse/Recurrence in Perinatal Women. Mindfulness. 2021;12(1):61-67.

96.Evans HEL, Forbes CC, Galvao DA, et al. Evaluating a web- and telephone-based personalised exercise intervention for individuals living with metastatic prostate cancer (ExerciseGuide): protocol for a pilot randomised controlled trial. Pilot Feasibility Stud. 2021;7(1):21.

97.Evans JMM, Irvine L, Connelly J, Cameron DM. The development of an intervention for diabetes prevention among people with impaired glucose regulation: feasibility and acceptability of an intervention component. Pilot and Feasibility Studies. 2019;5(1).

98.Ezeugwu VE, Manns PJ. Using Intervention Mapping to develop and implement a home-based sedentary behavior change intervention after stroke. Translational Behavioral Medicine. 2020;10(1):87-95.

99.Forbes G, Newton S, Calvete CC, et al. MEMPHIS: a smartphone app using psychological approaches for women with chronic pelvic pain presenting to gynaecology clinics: a randomised feasibility trial. Bmj Open. 2020;10(3).

100.Fortune J, Norris M, Stennett A, et al. 'I can do this': a qualitative exploration of acceptability and experiences of a physical activity behaviour change intervention in people with multiple sclerosis in the UK. Bmj Open. 2020;10(3).

101.Frantz I, Foran HM, Lachman JM, et al. Prevention of child mental health problems in Southeastern Europe: a multicentre sequential study to adapt, optimise and test the parenting programme 'Parenting for Lifelong Health for Young Children', protocol for stage 1, the feasibility study. Bmj Open. 2019;9(1).

102.Galea S, Salvaris CA, Yap MBH, Norton PJ, Lawrence KA. Feasibility and acceptability of an enhanced cognitive behavioural therapy programme for parent-child dyads with anxiety disorders: a mixed-methods pilot trial protocol. Pilot Feasibility Stud. 2021;7(1):109.

103.Gallyer V, Smith TO, Fordham B, et al. Getting Recovery Right After Neck Dissection (GRRAND-F): mixed-methods feasibility study to design a pragmatic randomised controlled trial protocol. BMJ Open. 2021;11(6):e045741.

104.Gammon C, Morton K, Atkin A, et al. Introducing physically active lessons in UK secondary schools: feasibility study and pilot cluster-randomised controlled trial. BMJ Open. 2019;9(5):e025080.

105.Geffen LN, Kelly G, Morris JN, Howard EP. Peer-to-peer support model to improve quality of life among highly vulnerable, low-income older adults in Cape Town, South Africa. Bmc Geriatrics. 2019;19(1).

106.Gellert P, Beyer AK, Tegeler C, et al. Outpatient psychotherapy for home-living vulnerable older adults with depression: study protocol of the PSY-CARE trial. BMC Geriatr. 2020;20(1):271.

107.Geraghty AWA, Stanford R, Stuart B, et al. Using an Internet intervention to support self-management of low back pain in primary care: findings from a randomised controlled feasibility trial (SupportBack). Bmj Open. 2018;8(3).

108.Gibson CA, Gupta A, Greene JL, Lee J, Mount RR, Sullivan DK. Feasibility and acceptability of a televideo physical activity and nutrition program for recent kidney transplant recipients. Pilot Feasibility Stud. 2020;6:126.

109.Gillespie J, Hughes A, Gibson AM, Haines J, Taveras E, Reilly JJ. Protocol for Healthy Habits Happy Homes (4H) Scotland: feasibility of a participatory approach to adaptation and implementation of a study aimed at early prevention of obesity. Bmj Open. 2019;9(6).

110.Gimbel RW, Rennert LM, Crawford P, et al. Enhancing Patient Activation and Self-Management Activities in Patients With Type 2 Diabetes Using the US Department of Defense Mobile Health Care Environment: Feasibility Study. J Med Internet Res. 2020;22(5):e17968.

111.Glenn J, Madero EN, Gray M, et al. Engagement With a Digital Platform for Multimodal Cognitive Assessment and Multidomain Intervention in a Japanese Population: Pilot, Quasi-Experimental, Longitudinal Study. JMIR Mhealth Uhealth. 2019;7(10):e15733.

112.Golla A. Home-based balance training using Wii Fit™: A pilot randomised controlled trial with mobile older stroke survivors. Pilot and Feasibility Studies. 2018.

113.Gonot-Schoupinsky FN, Garip G, Sheffield D, Omar OM, Arora T. Prescribing laughter to ameliorate mental health, sleep, and wellbeing in university students: A protocol for a feasibility study of a randomised controlled trial. Contemp Clin Trials Commun. 2020;20:100676.

114.Gonzalez-Santos J, Soto-Camara R, Rodriguez-Fernandez P, et al. Effects of home-based mirror therapy and cognitive therapeutic exercise on the improvement of the upper extremity functions in patients with severe hemiparesis after a stroke: a protocol for a pilot randomised clinical trial. BMJ Open. 2020;10(9):e035768.

115.Gorman JR, Drizin JH, Al-Ghadban FA, Rendle KA. Adaptation and feasibility of a multimodal mindfulness-based intervention to promote sexual health in cancer survivorship. Transl Behav Med. 2021.

116.Gosselin A, Carillon S, Coulibaly K, et al. Participatory development and pilot testing of the Makasi intervention: a community-based outreach intervention to improve sub-Saharan and Caribbean immigrants' empowerment in sexual health. BMC Public Health. 2019;19(1):1646.

117.Graham L, Ellwood A, Hull K, et al. A posture and mobility training package for care home staff: results of a cluster randomised controlled feasibility trial (the PATCH trial). Age Ageing. 2020;49(5):821-828.

118.Granic A, Hurst C, Dismore L, et al. Milk and resistance exercise intervention to improve muscle function in community-dwelling older adults at risk of sarcopenia (MIlkMAN): protocol for a pilot study. Bmj Open. 2019;9(10).

119.Greidanus MA, de Rijk AE, de Boer A, et al. A randomised feasibility trial of an employer-based intervention for enhancing successful return to work of cancer survivors (MiLES intervention). BMC Public Health. 2021;21(1):1433.

120.Griffith GM, Hastings RP, Williams J, et al. Mixed Experiences of a Mindfulness-Informed Intervention: Voices from People with Intellectual Disabilities, Their Supporters, and Therapists. Mindfulness. 2019;10(9):1828-1841.

121.Guagliano JM, Brown HE, Coombes E, et al. The development and feasibility of a randomised family-based physical activity promotion intervention: the Families Reporting Every Step to Health (FRESH) study. Pilot Feasibility Stud. 2019;5:21.

122.Guidetti S, Nielsen KT, von Bulow C, Pilegaard MS, Klokker L, Waehrens EE. Evaluation of an intervention programme addressing ability to perform activities of daily living among persons with chronic conditions: study protocol for a feasibility trial (ABLE). Bmj Open. 2018;8(5).

123.Guilamo-Ramos V, Bowman AS, Santa Maria D, Kabemba F, Geronimo Y. Addressing a Critical Gap in U.S. National Teen Pregnancy Prevention Programs: The Acceptability and Feasibility of Father-Based Sexual and Reproductive Health Interventions for Latino Adolescent Males. J Adolesc Health. 2018;62(3S):S81-S86.

124.Guo X, Ohsawa C, Suzuki A, Sekiyama K. Improved Digit Span in Children after a 6-Week Intervention of Playing a Musical Instrument: An Exploratory Randomized Controlled Trial. Front Psychol. 2017;8:2303.

125.Hadley R. """Dance Like Nobody's Watching"": Exploring the Role of Dance-Based Interventions in Perceived Well-Being and Bodily Awareness in People With Parkinson's". Frontiers in Psychology. 2020.

126.Hagelskjaer V, Nielsen KT, von Bulow C, Graff M, Waehrens EE. Occupational therapy addressing the ability to perform activities of daily living among persons living with chronic conditions: a randomised controlled pilot study of ABLE 2.0. Pilot and Feasibility Studies. 2021;7(1).

127.Haines M. "Feasibility of procedures for a randomised pilot study of reduced exertion, high-intensity interval training (REHIT) with non-diabetic hyperglycaemia patients". Pilot Feasibility Stud. 2020.

128.Hallett V. Introducing 'Predictive Parenting': A Feasibility Study of a New Group Parenting Intervention Targeting Emotional and Behavioral Difficulties in Children with Autism Spectrum Disorder. J Autism Dev Disord. 2021.

129.Han MK, Cho B, Kwon H, et al. A Mobile-Based Comprehensive Weight Reduction Program for the Workplace (Health-On): Development and Pilot Study. JMIR Mhealth Uhealth. 2019;7(11):e11158.

130.Hanrahan R, Smith E, Johnson H, Constantin A, Brosnan M. A Pilot Randomised Control Trial of Digitally-Mediated Social Stories for Children on the Autism Spectrum. J Autism Dev Disord. 2020;50(12):4243-4257.

131.Hansen P, Main C, Hartling L. Dance Intervention Affects Social Connections and Body Appreciation Among Older Adults in the Long Term Despite COVID-19 Social Isolation: A Mixed Methods Pilot Study. Front Psychol. 2021;12:635938.

132.Haramaki Y, Kabir RS, Abe K, Yoshitake T. Promoting Self-Regulatory Management of Chronic Pain Through Dohsa-hou: Single-Case Series of Low-Functioning Hemodialysis Patients. Front Psychol. 2019;10:1394.

133.Hargreaves EA, Haszard JJ, Shaw S, Peddie MC. Protocol for a pilot trial to assess the feasibility of the Move More @ Work intervention to encourage employees to take the opportunity to move (be physically active) after every 30 min of sitting. Pilot and Feasibility Studies. 2021;7(1).

134.Hawker CO, Merkouris SS, Youssef GJ, Dowling NA. A Smartphone-Delivered Ecological Momentary Intervention for Problem Gambling (GamblingLess: Curb Your Urge): Single-Arm Acceptability and Feasibility Trial. J Med Internet Res. 2021;23(3):e25786.

135.Hawkins J, Charles JM, Edwards M, et al. Acceptability and Feasibility of Implementing Accelorometry-Based Activity Monitors and a Linked Web Portal in an Exercise Referral Scheme: Feasibility Randomized Controlled Trial. J Med Internet Res. 2019;21(3):e12374.

136.Hawley-Hague H. Can smartphone technology be used to support an effective home exercise intervention to prevent falls amongst community dwelling older adults?: the TOGETHER feasibility RCT study protocol. BMJ Open. 2019.

137.Hayes C, Ciblis A, Darker C, et al. We Can Quit2 (WCQ2): a community-based intervention on smoking cessation for women living in disadvantaged areas of Ireland-study protocol for a pilot cluster randomised controlled trial. Pilot and Feasibility Studies. 2019;5(1).

138.Heinrich K, Sanchez K, Hui C, et al. Impact of an electronic medium delivery of warfarin education in a low income, minority outpatient population: a pilot intervention study. Bmc Public Health. 2019;19(1).

139.Hemenway M. Development of a mindfulness-based treatment for smoking cessation and the modification of alcohol use: A protocol for a randomized controlled trial and pilot study findings. Contemp Clin Trials. 2021.

140.Hendricks BA, Lofton C, Azuero A, et al. The project ENABLE Cornerstone randomized pilot trial: Protocol for lay navigator-led early palliative care for African-American and rural advanced cancer family caregivers. Contemp Clin Trials Commun. 2019;16:100485.

141.Heslehurst N, Rankin J, McParlin C, et al. GestationaL Obesity Weight management: Implementation of National Guidelines (GLOWING): a pilot cluster randomised controlled trial of a guideline implementation intervention for the management of maternal obesity by midwives. Pilot Feasibility Stud. 2018;4:47.

142.Hester KLM, Ryan V, Newton J, Rapley T, De Soyza A. Bronchiectasis Information and Education: a randomised, controlled feasibility trial. Trials. 2020;21(1).

143.Hill JL, Zoellner JM, You W, et al. Participatory development and pilot testing of iChoose: an adaptation of an evidence-based paediatric weight management program for community implementation. Bmc Public Health. 2019;19.

144.Hirvonen M, Purcell C, Elliott L, et al. Peer-to-Peer Sharing of Social Media Messages on Sexual Health in a School-Based Intervention: Opportunities and Challenges Identified in the STASH Feasibility Trial. J Med Internet Res. 2021;23(2):e20898.

145.Holler P, Jaunig J, Amort FM, et al. Holistic physical exercise training improves physical literacy among physically inactive adults: a pilot intervention study. Bmc Public Health. 2019;19.

146.Holliday R, Preshaw PM, Ryan V, et al. A feasibility study with embedded pilot randomised controlled trial and process evaluation of electronic cigarettes for smoking cessation in patients with periodontitis. Pilot Feasibility Stud. 2019;5:74.

147.Holloway A, Guthrie V, Waller G, et al. A two-arm parallel-group individually randomised prison pilot study of a male remand alcohol intervention for self-efficacy enhancement: the APPRAISE study protocol. BMJ Open. 2021;11(4):e040636.

148.Holmlund L, Guidetti S, Hultling C, Seiger A, Eriksson G, Asaba E. Evaluating the feasibility of ReWork-SCI: a person-centred intervention for return-to-work after spinal cord injury. BMJ Open. 2020;10(8):e036000.

149.Holt N. Is there a need for formal undergraduate patient handover training and could an educational workshop effectively provide this? A proof-of-concept study in a Scottish Medical School. BMJ Open. 2020.

150.Hopkins LC. "Feasibility and acceptability of technologybased caregiver engagement strategies delivered in a summertime childhood obesity prevention intervention: Results from an internal pilot of the Camp NERF (Nutrition, Education, Recreation, and Fitness) study". Pilot and Feasibility Studies. 2018.

151.Hopley C, Andrews E, Klem P, et al. Evaluating the feasibility of a pharmacist-guided patient-driven intervention to improve blood pressure control in patients with CKD. Pilot Feasibility Stud. 2019;5:23.

152.Howells K. Can a Community-Based Football Program Benefit Motor Ability in Children with Autism Spectrum Disorder? A Pilot Evaluation Considering the Role of Social Impairments. J Autism Dev Disord. 2021.

153.Hubbard G, Beeken RJ, Taylor C, et al. HALT (Hernia Active Living Trial): protocol for a feasibility study of a randomised controlled trial of a physical activity intervention to improve quality of life in people with bowel stoma with a bulge/parastomal hernia. Pilot Feasibility Stud. 2020;6:142.

154.Hubbard G, Cherrie J, Gray J, et al. Sun protection education for adolescents: a feasibility study of a wait-list controlled trial of an intervention involving a presentation, action planning, and SMS messages and using objective measurement of sun exposure. Bmc Public Health. 2020;20(1).

155.Huda TM, Alam A, Tahsina T, et al. Mobile-Based Nutrition Counseling and Unconditional Cash Transfers for Improving Maternal and Child Nutrition in Bangladesh: Pilot Study. JMIR Mhealth Uhealth. 2018;6(7):e156.

156.Hughes E, Mitchell N, Gascoyne S, et al. The RESPECT study: a feasibility randomised controlled trial of a sexual health promotion intervention for people with serious mental illness in community mental health services in the UK. BMC Public Health. 2020;20(1):1736.

157.Hughes RCE. "Prediabetes in pregnancy, can early intervention improve outcomes? A feasibility study for a parallel randomised clinical trial". BMJ Open. 2018.

158.Hui VK, Wong CY, Ma EK, Ho FY, Chan CS. Treating depression with a smartphone-delivered self-help cognitive behavioral therapy for insomnia: study protocol for a parallel group randomized controlled trial. Trials. 2020;21(1):843.

159.Ibanez GE, Fennie K, Larkey L, et al. A tai chi/qigong intervention for older adults living with HIV: a study protocol of an exploratory clinical trial. Trials. 2020;21(1):804.

160.Igwesi-Chidobe CN, Kitchen S, Sorinola IO, Godfrey EL. Evidence, theory and context: using intervention mapping in the development of a community-based self-management program for chronic low back pain in a rural African primary care setting-the good back program. Bmc Public Health. 2020;20(1).

161.Ingerski LM, Means B, Wang F, et al. Preventing Medication Nonadherence of Youth (13-24 Years) With HIV Initiating Antiretroviral Therapy. J Adolesc Health. 2021;69(4):644-652.

162.Irvine L. Design and development of a complex narrative intervention delivered by text messages to reduce binge drinking among socially disadvantaged men. Pilot Feasibility Stud. 2018.

163.Jacobs CA, Mace RA, Greenberg J, et al. Development of a mind body program for obese knee osteoarthritis patients with comorbid depression. Contemp Clin Trials Commun. 2021;21:100720.

164.Jacobs JM, Rapoport CS, Horenstein A, et al. Study protocol for a randomised controlled feasibility trial of a virtual intervention (STRIDE) for symptom management, distress and adherence to adjuvant endocrine therapy after breast cancer. Bmj Open. 2021;11(1).

165.Jacobsen P, Peters E, Robinson EJ, Chadwick P. Mindfulness-based crisis interventions (MBCI) for psychosis within acute inpatient psychiatric settings; a feasibility randomised controlled trial. Bmc Psychiatry. 2020;20(1).

166.Joag K, Shields-Zeeman L, Kapadia-Kundu N, Kawade R, Balaji M, Pathare S. Feasibility and acceptability of a novel community-based mental health intervention delivered by community volunteers in Maharashtra, India: the Atmiyata programme. Bmc Psychiatry. 2020;20(1).

167.Jobst S, Leppla L, Koberich S. A self-management support intervention for patients with atrial fibrillation: a randomized controlled pilot trial. Pilot Feasibility Stud. 2020;6:87.

168.Johri M, Chandra D, Kone KG, et al. Social and Behavior Change Communication Interventions Delivered Face-to-Face and by a Mobile Phone to Strengthen Vaccination Uptake and Improve Child Health in Rural India: Randomized Pilot Study. JMIR Mhealth Uhealth. 2020;8(9):e20356.

169.Jolly K, Ingram J, Clarke J, et al. Protocol for a feasibility trial for improving breast feeding initiation and continuation: assets-based infant feeding help before and after birth (ABA). Bmj Open. 2018;8(1).

170.Jones S, Robinson H, Riste L, et al. Integrated psychological therapy for people with bipolar disorder and co-morbid alcohol use: A feasibility and acceptability randomised controlled trial. Contemporary Clinical Trials Communications. 2018;10:193-198.

171.Joss D, Lazar SW, Teicher MH. Nonattachment Predicts Empathy, Rejection Sensitivity, and Symptom Reduction After a Mindfulness-Based Intervention Among Young Adults with a History of Childhood Maltreatment. Mindfulness. 2020;11(4):975-990.

172.Juszczyk D, Gillison F. Juicy June: a mass-participation snack-swap challenge-results from a mixed methods feasibility study. Pilot Feasibility Stud. 2018;4:119.

173.Kabugo D, Nakamura H, Magnusson B, et al. Mixed-method study to assess the feasibility, acceptability and early effectiveness of the Hospital to Home programme for follow-up of high-risk newborns in a rural district of Central Uganda: a study protocol. Bmj Open. 2021;11(3).

174.Kam JKM, Wong LK, Fu KCW. Creation of Sexually Transmitted Diseases Education Program for Young Adults in Rural Cambodia. Front Public Health. 2019;7:50.

175.Kane JC, Sharma A, Murray LK, et al. Efficacy of the Common Elements Treatment Approach (CETA) for Unhealthy Alcohol Use Among Adults with HIV in Zambia: Results from a Pilot Randomized Controlled Trial. Aids and Behavior. 2021.

176.Kaplan RL, El Khoury C, Wehbe S, Lize N, Mokhbat J. Pilot Results from the First HIV/AIDS Intervention Among Transgender Women in the Middle East: Gender Affirmation and Social Support from Within Trans Communities in Beirut, Lebanon. Aids Research and Human Retroviruses. 2020;36(6):501-512.

177.Karekla M, Savvides SN, Gloster A. An Avatar-Led Intervention Promotes Smoking Cessation in Young Adults: A Pilot Randomized Clinical Trial. Ann Behav Med. 2020;54(10):747-760.

178.Kaselionyte J. """It's a matter of building bridges..."" - feasibility of a carer involvement intervention for inpatients with severe mental illness". BMC Psychiatry. 2019.

179.Katz DA, Mott SL, Utech JA, et al. Time to put it out - nurse-facilitated tobacco treatment in a comprehensive cancer center. Transl Behav Med. 2021;11(9):1726-1738.

180.Kay MC, Burroughs J, Askew S, Bennett GG, Armstrong S, Steinberg DM. Digital Weight Loss Intervention for Parents of Children Being Treated for Obesity: A Prospective Cohort Feasibility Trial. J Med Internet Res. 2018;20(12):e11093.

181.Keene DJ, Costa ML, Tutton E, et al. Progressive functional exercise versus best practice advice for adults aged 50 years or over after ankle fracture: protocol for a pilot randomised controlled trial in the UK - the Ankle Fracture Treatment: Enhancing Rehabilitation (AFTER) study. BMJ Open. 2019;9(11):e030877.

182.Kelly JT, Warner MM, Conley M, et al. Feasibility and acceptability of telehealth coaching to promote healthy eating in chronic kidney disease: a mixed-methods process evaluation. BMJ Open. 2019;9(1):e024551.

183.Keng SL, Sahimi HBMS, Chan LF, et al. Implementation of brief dialectical behavior therapy skills training among borderline personality disorder patients in Malaysia: feasibility, acceptability, and preliminary outcomes. Bmc Psychiatry. 2021;21(1).

184.Kertesz M, Humphreys C, Larance LY, Vicary D, Spiteri-Staines A, Ovenden G. Working with women who use force: a feasibility study protocol of the Positive (+) SHIFT group work programme in Australia. Bmj Open. 2019;9(5).

185.Khidir H, Psaros C, Greener L, et al. Developing a Safer Conception Intervention for Men Living with HIV in South Africa. AIDS Behav. 2018;22(6):1725-1735.

186.Khoza N, Stadler J, MacPhail C, Chikandiwa A, Brahmbhatt H, Delany-Moretlwe S. Cash transfer interventions for sexual health: meanings and experiences of adolescent males and females in inner-city Johannesburg. BMC Public Health. 2018;18(1):120.

187.Kimber M, Harms S, Soreni N, et al. LIVES for families psychological first aid training programme to address COVID-19 psychological distress: a mixed methods acceptability and feasibility protocol. BMJ Open. 2021;11(5):e049995.

188.Kinnear FJ, Hamilton-Shield JP, Stensel DJ, et al. Nutrition and physical activity intervention for families with familial hypercholesterolaemia: protocol for a pilot randomised controlled feasibility study. Pilot Feasibility Stud. 2020;6:42.

189.Konig LM, Renner B. Boosting healthy food choices by meal colour variety: results from two experiments and a just-in-time Ecological Momentary Intervention. BMC Public Health. 2019;19(1):975.

190.Kramer F, Labudek S, Jansen CP, et al. Development of a conceptual framework for a group-based format of the Lifestyle-integrated Functional Exercise (gLiFE) programme and its initial feasibility testing. Pilot Feasibility Stud. 2020;6:6.

191.Kuhns LM, Hotton AL, Perloff J, et al. Evaluation of Translife Care: An Intervention to Address Social Determinants of Engagement in HIV Care Among Transgender Women of Color. Aids and Behavior. 2021;25(Suppl 1):13-19.

192.Kwon BC, VanDam C, Chiuve SE, et al. Improving Heart Disease Risk Through Quality-Focused Diet Logging: Pre-Post Study of a Diet Quality Tracking App. JMIR Mhealth Uhealth. 2020;8(12):e21733.

193.Labbe AK, Wilner JG, Coleman JN, et al. A qualitative study of the feasibility and acceptability of a smoking cessation program for people living with HIV and emotional dysregulation. AIDS Care. 2019;31(5):609-615.

194.Laddu D, Kim H, Phillips SA, Ma J. INERTIA: A pilot study of the impact of progressive resistance training on blood pressure control in older adults with sarcopenia. Contemp Clin Trials. 2021;108:106516.

195.Lambert JD, Greaves CJ, Farrand P, Price L, Haase AM, Taylor AH. Web-Based Intervention Using Behavioral Activation and Physical Activity for Adults With Depression (The eMotion Study): Pilot Randomized Controlled Trial. J Med Internet Res. 2018;20(7):e10112.

196.Lambert SD, Duncan LR, Ellis J, et al. A study protocol for a multicenter randomized pilot trial of a dyadic, tailored, web-based, psychosocial, and physical activity self-management program (TEMPO) for men with prostate cancer and their caregivers. Pilot and Feasibility Studies. 2021;7(1).

197.Lane C, Carson V, Morton K, et al. A real-world feasibility study of the PLAYshop: a brief intervention to facilitate parent engagement in developing their child's physical literacy. Pilot and Feasibility Studies. 2021;7(1).

198.Langford R, Jago R, White J, et al. A physical activity, nutrition and oral health intervention in nursery settings: process evaluation of the NAP SACC UK feasibility cluster RCT. Bmc Public Health. 2019;19.

199.Larsen B, Benitez T, Cano M, et al. Web-Based Physical Activity Intervention for Latina Adolescents: Feasibility, Acceptability, and Potential Efficacy of the Ninas Saludables Study. J Med Internet Res. 2018;20(5):e170.

200.Larsen LQ, Schnor H, Tersbol BP, Ebdrup BH, Nordsborg NB, Midtgaard J. The impact of exercise training complementary to early intervention in patients with first-episode psychosis: a qualitative sub-study from a randomized controlled feasibility trial. BMC Psychiatry. 2019;19(1):192.

201.Latchem-Hastings J, Randell E, Button K, et al. Lifestyle, exercise and activity package for people living with progressive multiple sclerosis (LEAP-MS): protocol for a single-arm feasibility study. Pilot and Feasibility Studies. 2021;7(1).

202.Lauwerier E, Van Poel E, Van der Veken K, Van Roy K, Willems S. Evaluation of a program targeting sports coaches as deliverers of health-promoting messages to at-risk youth: Assessing feasibility using a realist-informed approach. Plos One. 2020;15(9).

203.Lawlor ER, Cupples ME, Donnelly M, Tully MA. Promoting physical activity among community groups of older women in socio-economically disadvantaged areas: randomised feasibility study. Trials. 2019;20.

204.Lee MK, Kim NK, Jeon JY. Effect of the 6-week home-based exercise program on physical activity level and physical fitness in colorectal cancer survivors: A randomized controlled pilot study. PLoS One. 2018;13(4):e0196220.

205.Lehman WEK, Pankow J, Rowan GA, et al. StaySafe: A self-administered android tablet application for helping individuals on probation make better decisions pertaining to health risk behaviors. Contemporary Clinical Trials Communications. 2018;10:86-93.

206.Leightley D, Puddephatt JA, Jones N, et al. A Smartphone App and Personalized Text Messaging Framework (InDEx) to Monitor and Reduce Alcohol Use in Ex-Serving Personnel: Development and Feasibility Study. JMIR Mhealth Uhealth. 2018;6(9):e10074.

207.Lian YT, Zhao Y, Wang J, et al. A health communication intervention to integrate partner testing with antiretroviral therapy service among men who have sex with men in China: an observational cohort study. Bmc Public Health. 2018;18.

208.Lo SHS, Chau JPC, Choi KC, Yeung J, Li SH, Demers M. Feasibility of a ballet-inspired low-impact at-home workout programme for adults with stroke: a mixed-methods exploratory study protocol. BMJ Open. 2021;11(4):e045064.

209.Lock M, Post D, Dollman J, Parfitt G. Feasibility and Process Evaluation of a Need-Supportive Physical Activity Program in Aged Care Workers: The Activity for Well-Being Project. Front Psychol. 2020;11:518413.

210.Loeb DF, Monson SP, Lockhart S, et al. Mixed method evaluation of Relational Team Development (RELATED) to improve team-based care for complex patients with mental illness in primary care. BMC Psychiatry. 2019;19(1):299.

211.Loeckx M, Rabinovich RA, Demeyer H, et al. Smartphone-Based Physical Activity Telecoaching in Chronic Obstructive Pulmonary Disease: Mixed-Methods Study on Patient Experiences and Lessons for Implementation. JMIR Mhealth Uhealth. 2018;6(12):e200.

212.Lopez Segui F, Pratdepadua Bufill C, Abdon Gimenez N, Martinez Roldan J, Garcia Cuyas F. The Prescription of Mobile Apps by Primary Care Teams: A Pilot Project in Catalonia. JMIR Mhealth Uhealth. 2018;6(6):e10701.

213.Loucks EB, Nardi WR, Gutman R, et al. Mindfulness-Based Blood Pressure Reduction (MB-BP): Stage 1 single-arm clinical trial. PLoS One. 2019;14(11):e0223095.

214.Louwagie GM, Morojele N, Siddiqi K, et al. Addressing tobacco smoking and drinking to improve TB treatment outcomes, in South Africa: a feasibility study of the ProLife program. Translational Behavioral Medicine. 2020;10(6):1491-1503.

215.Luberto CM, Park ER, Goodman JH. Postpartum Outcomes and Formal Mindfulness Practice in Mindfulness-Based Cognitive Therapy for Perinatal Women. Mindfulness (N Y). 2018;9(3):850-859.

216.Machen L, Handley MA, Powe N, Tuot D. Engagement With a Health Information Technology-Augmented Self-Management Support Program in a Population With Limited English Proficiency: Observational Study. JMIR Mhealth Uhealth. 2021;9(5):e24520.

217.Maddison R, Hargreaves EA, Jiang YN, et al. Rugby Fans in Training New Zealand (RUFIT-NZ): protocol for a randomized controlled trial to assess the effectiveness and cost-effectiveness of a healthy lifestyle program for overweight men delivered through professional rugby clubs in New Zealand. Trials. 2020;21(1).

218.Malden S, Hughes AR, Gibson AM, et al. Adapting the ToyBox obesity prevention intervention for use in Scottish preschools: protocol for a feasibility cluster randomised controlled trial. Bmj Open. 2018;8(10).

219.Malins S, Owen R, Wright I, et al. Acceptance and commitment therapy for young brain tumour survivors: study protocol for an acceptability and feasibility trial. BMJ Open. 2021;11(6):e051091.

220.Mama SK, Bhuiyan N, Bopp MJ, McNeill LH, Lengerich EJ, Smyth JM. A faith-based mind-body intervention to improve psychosocial well-being among rural adults. Translational Behavioral Medicine. 2020;10(3):546-554.

221.Marshall S, Taki S, Love P, et al. Feasibility of a culturally adapted early childhood obesity prevention program among migrant mothers in Australia: a mixed methods evaluation. Bmc Public Health. 2021;21(1).

222.Martinez SA. Contingency management for smoking cessation among individuals with type 2 diabetes: protocol for a multi-center randomized controlled feasibility trial. Pilot Feasibility Stud. 2020.

223.Masulani-Mwale C, Kauye F, Gladstone M, Mathanga D. Development of a psycho-social intervention for reducing psychological distress among parents of children with intellectual disabilities in Malawi. Plos One. 2019;14(2).

224.Mattishent K, Lane K, Salter C, et al. Continuous glucose monitoring in older people with diabetes and memory problems: a mixed-methods feasibility study in the UK. BMJ Open. 2019;9(11):e032037.

225.Matvienko-Sikar K, Toomey E, Queally M, et al. Choosing Healthy Eating for Infant Health (CHErIsH) study: protocol for a feasibility study. BMJ Open. 2019;9(8):e029607.

226.Mayo-Wilson LJ, Coleman J, Timbo F, et al. Acceptability of a feasibility randomized clinical trial of a microenterprise intervention to reduce sexual risk behaviors and increase employment and HIV preventive practices (EMERGE) in young adults: a mixed methods assessment. Bmc Public Health. 2020;20(1).

227.Mazigo HD, Amuasi JH, Osei I, Kinung'hi SM. Integrating use of point-of-care circulating cathodic antigen rapid diagnostic tests by community health workers during mass drug administration campaigns to improve uptake of praziquantel treatment among the adult population at Kome Island, North-Western Tanzania: a cluster randomized community trial. Bmc Public Health. 2018;18.

228.McCool J, Tanielu H, Umali E, Whittaker R. Assessing the Cross-Cultural Adaptation and Translation of a Text-Based Mobile Smoking Cessation Program in Samoa (TXTTaofiTapaa): Pilot Study. JMIR Mhealth Uhealth. 2018;6(8):e173.

229.McCormack C, Kehoe B, Hardcastle SJ, et al. Pulmonary hypertension and home-based (PHAHB) exercise intervention: protocol for a feasibility study. BMJ Open. 2021;11(5):e045460.

230.McGovern R. Promoting Alcohol Reduction in Non-Treatment Seeking parents (PAReNTS): a protocol for a pilot feasibility cluster randomised controlled trial of alcohol screening and brief interventions to reduce parental alcohol use disorders in vulnerable families. Pilot Feasibility Stud. 2018.

231.McPherson AC, Biddiss E, Chen L, et al. Children and Teens in Charge of their Health (CATCH): A protocol for a feasibility randomised controlled trial of solution-focused coaching to foster healthy lifestyles in childhood disability. Bmj Open. 2019;9(3).

232.McVay MA, Yancy WS, Bennett GG, et al. A web-based intervention to increase weight loss treatment initiation: results of a cluster randomized feasibility and acceptability trial. Transl Behav Med. 2021;11(1):226-235.

233.Metcalfe RS, Atef H, Mackintosh K, et al. Time-efficient and computer-guided sprint interval exercise training for improving health in the workplace: a randomised mixed-methods feasibility study in office-based employees. BMC Public Health. 2020;20(1):313.

234.Midgley N, Besser SJ, Fearon P, Wyatt S, Byford S, Wellsted D. The Herts and Minds study: feasibility of a randomised controlled trial of Mentalization-Based Treatment versus usual care to support the wellbeing of children in foster care. BMC Psychiatry. 2019;19(1):215.

235.Mifsud JL, Stephenson J, Astin F, Galea J. Coronary risk reduction intervention for siblings and offspring of patients with premature coronary heart disease: the CRISO study protocol for a randomised controlled pilot study. Pilot Feasibility Stud. 2021;7(1):153.

236.Monin JK, Sperduto CM, Manigault AW, et al. Mindfulnes-Based Stress Reduction for Older Couples with Metabolic Syndrome: a Pilot Randomized Controlled Trial. Mindfulness (N Y). 2020;11(4):917-927.

237.Monroe AK, Pena JS, Moore RD, et al. Randomized controlled trial of a pictorial aid intervention for medication adherence among HIV-positive patients with comorbid diabetes or hypertension. Aids Care-Psychological and Socio-Medical Aspects of Aids/Hiv. 2018;30(2):199-206.

238.Monteiro-Guerra F, Signorelli GR, Tadas S, et al. A Personalized Physical Activity Coaching App for Breast Cancer Survivors: Design Process and Early Prototype Testing. JMIR Mhealth Uhealth. 2020;8(7):e17552.

239.Montgomery ET, Roberts ST, Reddy K, et al. Integration of a Relationship-focused Counseling Intervention with Delivery of the Dapivirine Ring for HIV Prevention to Women in Johannesburg: Results of the CHARISMA Pilot Study. AIDS Behav. 2021.

240.Moore SA. "A feasibility, acceptability and fidelity study of a multifaceted behaviour change intervention targeting free-living physical activity and sedentary behaviour in community dwelling adult stroke survivors". Pilot Feasibility Stud. 2020.

241.Moreno MA, Eickhoff J, Zhao QQ, Suris JC. College Students and Problematic Internet Use: A Pilot Study Assessing Self-Appraisal and Independent Behavior Change. Journal of Adolescent Health. 2019;64(1):131-133.

242.Morris AS, Murphy RC, Shepherd SO, Healy GN, Edwardson CL, Graves LEF. A multi-component intervention to sit less and move more in a contact centre setting: a feasibility study. BMC Public Health. 2019;19(1):292.

243.Morris RL, Gallacher K, Hann M, et al. Protocol for a non-randomised feasibility study evaluating a codesigned patient safety guide in primary care. BMJ Open. 2021;11(1):e039752.

244.Muhindo R, Mujugira A, Castelnuovo B, et al. Text message reminders and peer education increase HIV and Syphilis testing among female sex workers: a pilot quasi-experimental study in Uganda. Bmc Health Services Research. 2021;21(1).

245.Munakampe MN, Nkole T, Silumbwe A, Zulu JM, Cordero JP, Steyn PS. Feasibility testing of a community dialogue approach for promoting the uptake of family planning and contraceptive services in Zambia. BMC Health Serv Res. 2020;20(1):728.

246.Murphy ME, McSharry J, Byrne M, et al. Supporting care for suboptimally controlled type 2 diabetes mellitus in general practice with a clinical decision support system: a mixed methods pilot cluster randomised trial. BMJ Open. 2020;10(2):e032594.

247.Murray E, Daff K, Lavida A, Henley W, Irwin J, Valabhji J. Evaluation of the digital diabetes prevention programme pilot: uncontrolled mixed-methods study protocol. BMJ Open. 2019;9(5):e025903.

248.Musoke D, Namata C, Ndejjo R, Ssempebwa JC, Musoke MB. Integrated malaria prevention in rural communities in Uganda: a qualitative feasibility study for a randomised controlled trial. Pilot Feasibility Stud. 2021;7(1):155.

249.Naughton F, Brown C, High J, et al. Randomised controlled trial of a just-in-time adaptive intervention (JITAI) smoking cessation smartphone app: the Quit Sense feasibility trial protocol. BMJ Open. 2021;11(4):e048204.

250.Navarro-Haro MV, Modrego-Alarcon M, Hoffman HG, et al. Evaluation of a Mindfulness-Based Intervention With and Without Virtual Reality Dialectical Behavior Therapy((R)) Mindfulness Skills Training for the Treatment of Generalized Anxiety Disorder in Primary Care: A Pilot Study. Front Psychol. 2019;10:55.

251.Negm AM, Kennedy CC, Ioannidis G, et al. Getting fit for hip and knee replacement: a protocol for the Fit-Joints pilot randomized controlled trial of a multi-modal intervention in frail patients with osteoarthritis. Pilot Feasibility Stud. 2018;4:127.

252.Nelson KM, Perry NS, Stout CD, Dunsiger SI, Carey MP. The Young Men and Media Study: A Pilot Randomized Controlled Trial of a Community-Informed, Online HIV Prevention Intervention for 14-17-Year-Old Sexual Minority Males. Aids and Behavior. 2021.

253.Nestadt DF, Saisaengjan C, McKay MM, et al. CHAMP plus Thailand: Pilot Randomized Control Trial of a Family-Based Psychosocial Intervention for Perinatally HIV-Infected Early Adolescents. Aids Patient Care and Stds. 2019;33(5):227-236.

254.Newlands RSN, Ntessalen M, Clark J, et al. Pilot randomised controlled trial of Weight Watchers (R) referral with or without dietitian-led group support for weight loss in women treated for breast cancer: the BRIGHT (BReast cancer weIGHT loss) trial. Pilot and Feasibility Studies. 2019;5(1).

255.Ng WM, Cheung K. A feasibility study of a WhatsApp-delivered Transtheoretical Model-based intervention to promote healthy eating habits for firefighters in Hong Kong: a cluster randomized controlled trial. Trials. 2020;21(1):518.

256.Ngai JTK, Yu RWM, Chau KKY, Wong PWC. Effectiveness of a school-based programme of animal-assisted humane education in Hong Kong for the promotion of social and emotional learning: A quasi-experimental pilot study. PLoS One. 2021;16(3):e0249033.

257.Nicol GE, Kolko RP, Lenze EJ, et al. Change in Adiposity, Hepatic Triglyceride, and Carotid Intima Media Thickness during Behavioral Weight-Loss Treatment in Antipsychotic-Treated Youth. Journal of the American Academy of Child and Adolescent Psychiatry. 2018;57(10):S179-S179.

258.Nicolson GH, Hayes C, Darker C. A theory-based multicomponent intervention to reduce occupational sedentary behaviour in professional male workers: protocol for a cluster randomised crossover pilot feasibility study. Pilot Feasibility Stud. 2020;6(1):175.

259.Nielsen KT, Guidetti S, von Bulow C, Klokker L, Waehrens EE. Feasibility of ABLE 1.0-a program aiming at enhancing the ability to perform activities of daily living in persons with chronic conditions. Pilot and Feasibility Studies. 2021;7(1).

260.Nixon AC, Bampouras TM, Gooch HJ, et al. Home-based exercise for people living with frailty and chronic kidney disease: A mixed-methods pilot randomised controlled trial. PLoS One. 2021;16(7):e0251652.

261.Njau B, Lisasi E, Damian DJ, Mushi DL, Boulle A, Mathews C. Feasibility of an HIV self-testing intervention: a formative qualitative study among individuals, community leaders, and HIV testing experts in northern Tanzania. Bmc Public Health. 2020;20(1).

262.Njuguna IN, Beima-Sofie K, Mburu CW, et al. Adolescent transition to adult care for HIV-infected adolescents in Kenya (ATTACH): study protocol for a hybrid effectiveness-implementation cluster randomised trial. BMJ Open. 2020;10(12):e039972.

263.Nomikos PA, Hall M, Fuller A, et al. Fidelity assessment of nurse-led non-pharmacological package of care for knee pain in the package development phase of a feasibility randomised controlled trial based in secondary care: a mixed methods study. BMJ Open. 2021;11(7):e045242.

264.Odukoya OO. "Body, Soul and Spirit, an adaptation of two evidence-based interventions to promote physical activity and healthy eating among adults in churches in Lagos Nigeria: a three-arm cluster randomized controlled pilot trial". Pilot Feasibility Stud. 2020.

265.Olotu C, Lebherz L, Harter M, et al. Improvement of perioperative care of the elderly patient (PeriAge): protocol of a controlled interventional feasibility study. BMJ Open. 2019;9(11):e031837.

266.Oluwoye O, Dyck D, McPherson SM, et al. Developing and implementing a culturally informed FAmily Motivational Engagement Strategy (FAMES) to increase family engagement in first episode psychosis programs: mixed methods pilot study protocol. BMJ Open. 2020;10(8):e036907.

267.O'Regan A, Glynn L, Garcia Bengoechea E, et al. An evaluation of an intervention designed to help inactive adults become more active with a peer mentoring component: a protocol for a cluster randomised feasibility trial of the Move for Life programme. Pilot Feasibility Stud. 2019;5:88.

268.Park VT. Motivation to Participate in Precision Health Research and Acceptability of Texting as a Recruitment and Intervention Strategy Among Vietnamese Americans: Qualitative Study. JMIR Mhealth Uhealth. 2021.

269.Parkes T, Matheson C, Carver H, et al. Supporting Harm Reduction through Peer Support (SHARPS): testing the feasibility and acceptability of a peer-delivered, relational intervention for people with problem substance use who are homeless, to improve health outcomes, quality of life and social functioning and reduce harms: study protocol. Pilot and Feasibility Studies. 2019;5(1).

270.Parretti HM, Ives NJ, Tearne S, et al. Protocol for the feasibility and acceptability of a brief routine weight management intervention for postnatal women embedded within the national child immunisation programme: randomised controlled cluster feasibility trial with nested qualitative study (PIMMS-WL). BMJ Open. 2020;10(2):e033027.

271.Parry M, Dhukai A, Clarke H, et al. Development and usability testing of HEARTPAfemale symbolN: protocol for a mixed methods strategy to develop an integrated smartphone and web-based intervention for women with cardiac pain. BMJ Open. 2020;10(3):e033092.

272.Passaro RC, Chavez-Gomez S, Castaneda-Huaripata A, et al. Personalized Cognitive Counseling Reduces Drinking Expectancy Among Men Who Have Sex with Men and Transgender Women in Lima, Peru: A Pilot Randomized Controlled Trial. Aids and Behavior. 2020;24(11):3205-3214.

273.Patomella AH, Guidetti S, Malstam E, et al. Primary prevention of stroke: randomised controlled pilot trial protocol on engaging everyday activities promoting health. BMJ Open. 2019;9(11):e031984.

274.Payne P, Fiering S, Zava D, et al. Digital Delivery of Meditative Movement Training Improved Health of Cigarette-Smoke-Exposed Subjects. Front Public Health. 2018;6:282.

275.Pellecchia M, Beidas RS, Mandell DS, Cannuscio CC, Dunst CJ, Stahmer AC. Parent empowerment and coaching in early intervention: study protocol for a feasibility study. Pilot Feasibility Stud. 2020;6:22.

276.Pellet J, Weiss M, Zuniga F, Mabire C. Implementation and preliminary testing of a theory-guided nursing discharge teaching intervention for adult inpatients aged 50 and over with multimorbidity: a pragmatic feasibility study protocol. Pilot Feasibility Stud. 2021;7(1):71.

277.Pennington L, Stamp E, Smith J, et al. Internet delivery of intensive speech and language therapy for children with cerebral palsy: a pilot randomised controlled trial. BMJ Open. 2019;9(1):e024233.

278.Pergolizzi D, Crespo I, Balaguer A, et al. Proactive and systematic multidimensional needs assessment in patients with advanced cancer approaching palliative care: a study protocol. BMJ Open. 2020;10(2):e034413.

279.Piau A, Nourhashemi F, De Mauleon A, et al. Telemedicine for the management of neuropsychiatric symptoms in long- term care facilities: the DETECT study, methods of a cluster randomised controlled trial to assess feasibility. Bmj Open. 2018;8(6).

280.Picariello F, Moss-Morris R, Macdougall IC, et al. Cognitive-behavioural therapy (CBT) for renal fatigue (BReF): a feasibility randomised-controlled trial of CBT for the management of fatigue in haemodialysis (HD) patients. BMJ Open. 2018;8(3):e020842.

281.Pillay B, Ftanou M, Ritchie D, et al. Study protocol of a pilot study evaluating feasibility and acceptability of a psychosexual intervention for couples postallogeneic haematopoietic stem cell transplantation. BMJ Open. 2020;10(10):e039300.

282.Pinzon MM. """Pisando Fuerte"": an evidence-based falls prevention program for Hispanic/Latinos older adults: results of an implementation trial". BMC Geriatr. 2019.

283.Plantinga LC, Jones B, Johnson J, et al. Delivery of a patient-friendly functioning report to improve patient-centeredness of dialysis care: a pilot study. BMC Health Serv Res. 2019;19(1):891.

284.Porter KJ, Brock DJ, Estabrooks PA, et al. SIPsmartER delivered through rural, local health districts: adoption and implementation outcomes. Bmc Public Health. 2019;19(1).

285.Powell-Chandler A, Rees B, Broad C, et al. Physiotherapy and Anterior Resection Syndrome (PARiS) trial: feasibility study protocol. BMJ Open. 2018;8(6):e021855.

286.Puka K, Bax K, Andrade A, et al. A live-online mindfulness-based intervention for children living with epilepsy and their families: protocol for a randomized controlled trial of Making Mindfulness Matter (c). Trials. 2020;21(1).

287.Puttkammer N. An EMR-Based Alert with Brief Provider-Led ART Adherence Counseling: Promising Results of the InfoPlus Adherence Pilot Study Among Haitian Adults with HIV Initiating ART. AIDS Behav. 2020.

288.Quested E, Kwasnicka D, Thogersen-Ntoumani C, et al. Protocol for a gender-sensitised weight loss and healthy living programme for overweight and obese men delivered in Australian football league settings (Aussie-FIT): A feasibility and pilot randomised controlled trial. BMJ Open. 2018;8(10):e022663.

289.Quinones MM, Lombard-Newell J, Sharp D, Way V, Cross W. Case study of an adaptation and implementation of a Diabetes Prevention Program for individuals with serious mental illness. Transl Behav Med. 2018;8(2):195-203.

290.Rantanen T, Pynnonen K, Saajanaho M, et al. Individualized counselling for active aging: protocol of a single-blinded, randomized controlled trial among older people (the AGNES intervention study). Bmc Geriatrics. 2019;19.

291.Rees C, Craigie M, Slatyer S, et al. Mindful Self-Care and Resiliency (MSCR): protocol for a pilot trial of a brief mindfulness intervention to promote occupational resilience in rural general practitioners. BMJ Open. 2018;8(6):e021027.

292.Ribeiro DC, Jafarian Tangrood Z, Sole G, Abbott JH. Effectiveness of a tailored rehabilitation versus standard strengthening programme for patients with shoulder pain: a protocol for a feasibility randomised controlled trial (the Otago MASTER trial). BMJ Open. 2019;9(7):e028261.

293.Ringen PA, Falk RS, Antonsen B, et al. Using motivational techniques to reduce cardiometabolic risk factors in long term psychiatric inpatients: a naturalistic interventional study. Bmc Psychiatry. 2018;18.

294.Rivard M, Mello C, Mestari Z, et al. Using Prevent Teach Reinforce for Young Children to Manage Challenging Behaviors in Public Specialized Early Intervention Services for Autism. Journal of Autism and Developmental Disorders. 2021;51(11):3970-3988.

295.Robinson GW, Lee E, Silburn SR, Nagel P, Leckning B, Midford R. School-Based Prevention in Very Remote Settings: A Feasibility Trial of Methods and Measures for the Evaluation of a Social Emotional Learning Program for Indigenous Students in Remote Northern Australia. Front Public Health. 2020;8:552878.

296.Roy A, Druker S, Hoge EA, Brewer JA. Physician Anxiety and Burnout: Symptom Correlates and a Prospective Pilot Study of App-Delivered Mindfulness Training. JMIR Mhealth Uhealth. 2020;8(4):e15608.

297.Ruegg N, Moritz S, Berger T, Ludtke T, Westermann S. An internet-based intervention for people with psychosis (EviBaS): study protocol for a randomized controlled trial. BMC Psychiatry. 2018;18(1):102.

298.Said CM, Delahunt M, Hardidge A, et al. Recumbent cycling to improve outcomes in people with hip fracture: a feasibility randomized trial. BMC Geriatr. 2021;21(1):394.

299.Saidi F, Mutale W, Freeborn K, et al. Combination adherence strategy to support HIV antiretroviral therapy and pre-exposure prophylaxis adherence during pregnancy and breastfeeding: protocol for a pair of pilot randomised trials. BMJ Open. 2021;11(6):e046032.

300.Sangraula M. Protocol for a feasibility study of groupbased focused psychosocial support to improve the psychosocial well-being and functioning of adults affected by humanitarian crises in Nepal: Group Problem Management plus (PM+). Pilot and Feasibility Studies. 2018.

301.Scheerman JFM, van Empelen P, van Loveren C, van Meijel B. A Mobile App (WhiteTeeth) to Promote Good Oral Health Behavior Among Dutch Adolescents with Fixed Orthodontic Appliances: Intervention Mapping Approach. JMIR Mhealth Uhealth. 2018;6(8):e163.

302.Schueller SM, Glover AC, Rufa AK, et al. A Mobile Phone-Based Intervention to Improve Mental Health Among Homeless Young Adults: Pilot Feasibility Trial. JMIR Mhealth Uhealth. 2019;7(7):e12347.

303.Scudamore T, Liem A, Wiener M, et al. Mindful Melody: feasibility of implementing music listening on an inpatient psychiatric unit and its relation to the use of as needed medications for acute agitation. Bmc Psychiatry. 2021;21(1).

304.Sebastiao E, McAuley E, Shigematsu R, Motl RW. Feasibility study design and methods for a home-based, square-stepping exercise program among older adults with multiple sclerosis: The SSE-MS project. Contemporary Clinical Trials Communications. 2017;7:200-207.

305.Sexton JB, Adair KC. Forty-five good things: a prospective pilot study of the Three Good Things well-being intervention in the USA for healthcare worker emotional exhaustion, depression, work-life balance and happiness. Bmj Open. 2019;9(3).

306.Shack AR. "Improved Self-Esteem in Artists After Participating in the ""Building Confidence and Self-Esteem Toolbox Workshop""". Frontiers in Psychology. 2018.

307.Shahmanesh M, Okesola N, Chimbindi N, et al. Thetha Nami: participatory development of a peer-navigator intervention to deliver biosocial HIV prevention for adolescents and youth in rural South Africa. BMC Public Health. 2021;21(1):1393.

308.Sharma S, Jensen MP, Moseley GL, Abbott JH. Pain education for patients with non-specific low back pain in Nepal: protocol of a feasibility randomised clinical trial (PEN-LBP Trial). BMJ Open. 2018;8(8):e022423.

309.Shdaifat AA. "Pilot study to build capacity for family medicine with abbreviated, low-cost training programme with minimal impact on patient care for a cohort of 84 general practitioners caring for Palestinian refugees in Jordan". BMJ Open. 2019.

310.Short CE, Finlay A, Sanders I, Maher C. Development and pilot evaluation of a clinic-based mHealth app referral service to support adult cancer survivors increase their participation in physical activity using publicly available mobile apps. Bmc Health Services Research. 2018;18.

311.Shum AK, Lai ES, Leung WG, et al. A Digital Game and School-Based Intervention for Students in Hong Kong: Quasi-Experimental Design. J Med Internet Res. 2019;21(4):e12003.

312.Silverberg ND, Panenka WJ, Lizotte PP, Bayley MT, Dance D, Li LC. Promoting early treatment for mild traumatic brain injury in primary care with a guideline implementation tool: a pilot cluster randomised trial. BMJ Open. 2020;10(10):e035527.

313.Simoes MDMP, Gonze BD, Proenca NL, et al. Use of a smartphone app combined with gamification to increase the level of physical activity of adults and older adults: protocol of a sequential multiple assignment randomized trial. Trials. 2019;20(1).

314.Simons M. A web-based educational intervention to implement trauma-informed care in a paediatric healthcare setting: protocol for a feasibility study using pre-post mixed methods design. Pilot Feasibility Stud. 2020.

315.Sinclair KA, Zamora-Kapoor A, Townsend-Ing C, McElfish PA, Kaholokula JK. Implementation outcomes of a culturally adapted diabetes self-management education intervention for Native Hawaiians and Pacific islanders. BMC Public Health. 2020;20(1):1579.

316.Sisson SB, Salvatore AL, Hildebrand D, et al. Interventions to promote healthy environments in family child care homes in Oklahoma-Happy Healthy Homes: study protocol for a randomized controlled trial. Trials. 2019;20(1):541.

317.Slatyer S, Craigie M, Rees C, Davis S, Dolan T, Hegney D. Nurse Experience of Participation in a Mindfulness-Based Self-Care and Resiliency Intervention. Mindfulness. 2018;9(2):610-617.

318.Slining M, Wills S, Fair M, et al. LiveWell in early childhood: results from a two-year pilot intervention to improve nutrition and physical activity policies, systems and environments among early childhood education programs in South Carolina. Bmc Public Health. 2021;21(1).

319.Sluggett JK, Page AT, Chen EYH, et al. Protocol for a non-randomised pilot and feasibility study evaluating a multicomponent intervention to simplify medication regimens for people receiving community-based home care services. BMJ Open. 2019;9(7):e025345.

320.Smith LR, Amico KR, Fisher JD, Cunningham CO. 60 Minutes for health: examining the feasibility and acceptability of a low-resource behavioral intervention designed to promote retention in HIV care. AIDS Care. 2018;30(2):255-265.

321.Sommer J, Chung C, Haller DM, Pautex S. Shifting palliative care paradigm in primary care from better death to better end-of-life: a Swiss pilot study. BMC Health Serv Res. 2021;21(1):629.

322.Song MK, Ward SE, Hepburn K, Paul S, Shah RC, Morhardt DJ. SPIRIT advance care planning intervention in early stage dementias: An NIH stage I behavioral intervention development trial. Contemp Clin Trials. 2018;71:55-62.

323.Sremanakova J, Sowerbutts AM, Todd C, Cooke R, Burden S. Healthy Eating and Active Lifestyle After Bowel Cancer (HEAL ABC): feasibility randomised controlled trial protocol. Pilot Feasibility Stud. 2020;6(1):176.

324.Stamp E. Contamination within trials of community-based public health interventions: lessons from the HENRY feasibility study. Pilot Feasibility Stud. 2021.

325.Stephenson A, Garcia-Constantino M, Murphy MH, McDonough SM, Nugent CD, Mair JL. The "Worktivity" mHealth intervention to reduce sedentary behaviour in the workplace: a feasibility cluster randomised controlled pilot study. Bmc Public Health. 2021;21(1).

326.Stern M. "Design and rationale for ADAPT+: Optimizing an intervention to promote healthy behaviors in rural, Latino youth with obesity and their parents, using mindfulness strategies". Contemp Clin Trials. 2021.

327.Stone N. Enhancing condom use experiences among young men to improve correct and consistent condom use: feasibility of a home-based intervention strategy (HIS-UK). Pilot Feasibility Stud. 2018.

328.Stork MJ, Bell EG, Jung ME. Examining the Impact of a Mobile Health App on Functional Movement and Physical Fitness: Pilot Pragmatic Randomized Controlled Trial. JMIR Mhealth Uhealth. 2021;9(5):e24076.

329.Swift DL, McGee JE, Huff AC, et al. Prescribed exercise to Reduce Recidivism After Weight Loss-Pilot (PREVAIL-P): Design, methods and rationale. Contemp Clin Trials Commun. 2021;21:100717.

330.Swindle T, Martinez A, Borsheim E, Andres A. Adaptation of an exercise intervention for pregnant women to community-based delivery: a study protocol. BMJ Open. 2020;10(9):e038582.

331.Sylvetsky AC, Blake EF, Visek AJ, et al. Feasibility and acceptability of a randomized controlled trial to investigate withdrawal symptoms in response to caffeinated sugary drink cessation among children. Contemp Clin Trials Commun. 2021;22:100791.

332.Tabak RG. Pilot test of an interactive obesity treatment approach among employed adults in a university medical billing office. Pilot Feasibility Stud. 2020.

333.Tagalidou N, Distlberger E, Loderer V, Laireiter AR. Efficacy and feasibility of a humor training for people suffering from depression, anxiety, and adjustment disorder: a randomized controlled trial. Bmc Psychiatry. 2019;19.

334.Tang CM, Raat H, Yan MX, et al. Application of the health action process approach model for reducing excessive internet use behaviors among rural adolescents in China: a school-based intervention pilot study. Bmc Public Health. 2021;21(1).

335.Tang D, Mitchell P, Flood V, et al. Dietary intervention in patients with age-related macular degeneration: protocol for a randomised controlled trial. BMJ Open. 2019;9(2):e024774.

336.Tang JSY. Development and Feasibility of MindChip: A Social Emotional Telehealth Intervention for Autistic Adults. J Autism Dev Disord. 2021.

337.Taraldsen K, Mikolaizak AS, Maier AB, et al. Protocol for the PreventIT feasibility randomised controlled trial of a lifestyle-integrated exercise intervention in young older adults. BMJ Open. 2019;9(3):e023526.

338.Tarrant M, Carter M, Dean SG, et al. Singing for people with aphasia (SPA): results of a pilot feasibility randomised controlled trial of a group singing intervention investigating acceptability and feasibility. BMJ Open. 2021;11(1):e040544.

339.Taylor JL, Pezzimenti F, Burke MM, DaWalt LS, Lee CE, Rabideau C. Development, Feasibility, and Acceptability of a Nationally Relevant Parent Training to Improve Service Access During the Transition to Adulthood for Youth with ASD. Journal of Autism and Developmental Disorders. 2021.

340.Tebb KP, Leng Trieu S, Rico R, Renteria R, Rodriguez F, Puffer M. A Mobile Health Contraception Decision Support Intervention for Latina Adolescents: Implementation Evaluation for Use in School-Based Health Centers. JMIR Mhealth Uhealth. 2019;7(3):e11163.

341.Thogersen-Ntoumani C, Quested E, Smith BS, et al. Feasibility and preliminary effects of a peer-led motivationally-embellished workplace walking intervention: A pilot cluster randomized trial (the START trial). Contemp Clin Trials. 2020;91:105969.

342.Thomas DSK, Bull S, Nyanza EC, Hampanda K, Liedtke M, Ngallaba SE. An mHealth pilot designed to increase the reach of prevention of mother-to-child transmission of HIV (PMTCT) across the treatment cascade in a resource-constrained setting in Tanzania. PLoS One. 2019;14(2):e0212305.

343.Thomas ED, Zohura F, Hasan MT, et al. Formative research to scale up a handwashing with soap and water treatment intervention for household members of diarrhea patients in health facilities in Dhaka, Bangladesh (CHoBI7 program). Bmc Public Health. 2020;20(1).

344.Thompson D, Callender C, Gonynor C, et al. Using Relational Agents to Promote Family Communication Around Type 1 Diabetes Self-Management in the Diabetes Family Teamwork Online Intervention: Longitudinal Pilot Study. J Med Internet Res. 2019;21(9):e15318.

345.Toivonen HM, Hassandra M, Wright PM, et al. Feasibility of a Responsibility-Based Leadership Training Program for Novice Physical Activity Instructors. Front Psychol. 2021;12:648235.

346.Towe SL. Web-Based Cognitive Training to Improve Working Memory in Persons with Co-Occurring HIV Infection and Cocaine Use Disorder: Outcomes from a Randomized Controlled Trial. AIDS Behav. 2021.

347.Treacy S, Haggith A, Wickramasinghe ND, Van Bortel T. Dementia-friendly prisons: a mixed-methods evaluation of the application of dementia-friendly community principles to two prisons in England. BMJ Open. 2019;9(8):e030087.

348.Tsakos G, Brocklehurst PR, Watson S, et al. Improving the oral health of older people in care homes (TOPIC): a protocol for a feasibility study. Pilot Feasibility Stud. 2021;7(1):138.

349.Tull K. """Strong Teeth""-a study protocol for an early-phase feasibility trial of a complex oral health intervention delivered by dental teams to parents of young children". Pilot Feasibility Stud. 2019.

350.Tully MA, Cunningham C, Cupples ME, et al. Walk with Me: a protocol for a pilot RCT of a peer-led walking programme to increase physical activity in inactive older adults. Pilot Feasibility Stud. 2018;4:117.

351.Turan JM, Darbes LA, Musoke PL, et al. Development and Piloting of a Home-Based Couples Intervention During Pregnancy and Postpartum in Southwestern Kenya. AIDS Patient Care STDS. 2018;32(3):92-103.

352.Valenstein-Mah H, Simpson TL, Bowen S, et al. Feasibility pilot of a brief mindfulness intervention for college students with posttraumatic stress symptoms and problem drinking. Mindfulness (N Y). 2019;10(7):1255-1268.

353.Vetrova MV, Aleksandrova OV, Paschenko AE, et al. Early stages of HIV treatment cascade in people living with HIV in Saint-Petersburg, Russia. AIDS Care. 2018;30(7):857-862.

354.Voils CI, Pendergast J, Hale SL, et al. A randomized feasibility pilot trial of a financial incentives intervention for dietary self-monitoring and weight loss in adults with obesity. Transl Behav Med. 2021;11(4):954-969.

355.Vu L, Tun W, Apicella L, et al. Community-based antiretroviral therapy (ART) delivery for female sex workers in Tanzania: intervention model and baseline findings. AIDS Care. 2020;32(6):729-734.

356.Waelde LC, Hechanova MRM, Ramos PAP, Macia KS, Moschetto JM. Mindfulness and Mantra Training for Disaster Mental Health Workers in the Philippines. Mindfulness. 2018;9(4):1181-1190.

357.Wagner B, Latimer J, Adams E, et al. School-based intervention to address self-regulation and executive functioning in children attending primary schools in remote Australian Aboriginal communities. PLoS One. 2020;15(6):e0234895.

358.Wallbank G, Sherrington C, Hassett L, et al. Active Women over 50 online information and support to promote physical activity behaviour change: study protocol for a pilot trial. Pilot Feasibility Stud. 2020;6:91.

359.Wang GY, Taylor T, Sumich A, Krageloh C, Lee CQ, Siegert RJ. Cognitive Effect Following a Blended (Face to Face and Videoconference-Delivered) Format Mindfulness Training. Front Psychol. 2021;12:701459.

360.Wang H, Blake H, Chattopadhyay K. Development of a School-Based Intervention to Increase Physical Activity Levels Among Chinese Children: A Systematic Iterative Process Based on Behavior Change Wheel and Theoretical Domains Framework. Front Public Health. 2021;9:610245.

361.Watts P, Rance S, McGowan V, et al. The long-term health and wellbeing impacts of Healthy New Towns (HNTs): protocol for a baseline and feasibility study of HNT demonstrator sites in England. Pilot Feasibility Stud. 2020;6:4.

362.Wen X, Eiden RD, Justicia-Linde FE, et al. A multicomponent behavioral intervention for smoking cessation during pregnancy: a nonconcurrent multiple-baseline design. Transl Behav Med. 2019;9(2):308-318.

363.Whittemore R, Vilar-Compte M, Burrola-Mendez S, et al. Development of a diabetes self-management + mHealth program: tailoring the intervention for a pilot study in a low-income setting in Mexico. Pilot Feasibility Stud. 2020;6:25.

364.Wilchesky M, Mueller G, Morin M, et al. The OptimaMed intervention to reduce inappropriate medications in nursing home residents with severe dementia: results from a quasi-experimental feasibility pilot study. BMC Geriatr. 2018;18(1):204.

365.Williams J, Stubbs B, Richardson S, et al. 'Walk this way': results from a pilot randomised controlled trial of a health coaching intervention to reduce sedentary behaviour and increase physical activity in people with serious mental illness. Bmc Psychiatry. 2019;19(1).

366.Wrapson W, Dorrestein M, Wrapson J, et al. A Feasibility Study of a One-to-One Mindfulness-Based Intervention for Improving Mood in Stroke Survivors. Mindfulness. 2021;12(5):1148-1158.

367.Young J, Green J, Farrin A, et al. A multicentre, pragmatic, cluster randomised, controlled feasibility trial of the POD system of care. Age and Ageing. 2020;49(4):640-647.
